# A model of brain function to predict tau across Alzheimer’s disease and related disorders

**DOI:** 10.1038/s41467-026-78300-7

**Published:** 2026-10-02

**Authors:** Nick Corriveau-Lecavalier, Gemeng Zhang, Hugo Botha, Leland R. Barnard, Heather J. Wiste, Ellen Dicks, Venkatsampath R. Gogineni, Jeyeon Lee, Irene Sintini, Matthew L. Senjem, Christopher G. Schwarz, Petrice M. Cogswell, Derek R. Johnson, Jeffrey L. Gunter, Michael G. Kamykowski, Jonathan Graff-Radford, Vijay K. Ramanan, Julie A. Fields, Mary M. Machulda, Nikki H. Stricker, Bradley F. Boeve, Val J. Lowe, Kejal Kantarci, David S. Knopman, Jennifer L. Whitwell, Keith A. Josephs, Ronald C. Petersen, Clifford R. Jack, David T. Jones, or the Alzheimer’s Disease Neuroimaging Initiative

**Affiliations:** 1 Department of Neurology, Mayo Clinic Rochester, Rochester, MN USA; 2 Department of Psychiatry and Psychology, Mayo Clinic Rochester, Rochester, MN USA; 3 Department of Quantitative Health Sciences, Mayo Clinic Rochester, Rochester, MN USA; 4 Department of Neurology, Amsterdam UMC, Amsterdam, The Netherlands; 5 Department of Radiology, Mayo Clinic Rochester, Rochester, MN USA; 6 Department of Biomedical Engineering, College of Medicine, Hanyang University, Seoul, South Korea; 7 Department of Information Technology, Mayo Clinic Rochester, Rochester, MN USA

**Keywords:** Network models, Alzheimer's disease

## Abstract

We developed a machine learning framework grounded in a computational model of brain function and FDG-PET imaging to predict Alzheimer’s disease (AD)-type tau across AD and related disorders (ADRD). The model was trained on 1088 research participants spanning normal aging and ADRD with FDG-PET and flortaucipir-PET (FTP-PET). Validation was conducted in: (1) a longitudinal subset of the training cohort (*n* = 215), (2) Alzheimer’s Disease Neuroimaging Initiative (*n* = 295), and (3) a heretogeneous clinical cohort (*n* = 264; *n* = 28 with FTP-PET). Results showed that the model accurately predicted FTP-PET when applied at the participant-level (*R*^*2*^ ~ 0.34-0.71). It performed well in clinical scenarios, including classification of low versus high tau stages (79–95% accuracy) and prediction of tau in a heterogeneous clinical cohort without FTP-PET. These findings advance the understanding of the relationship between neurodegeneration and AD-type tau and provide a framework for estimating AD-type tau across ADRD.

## Abstract (web summary)

Researchers developed a machine learning model using FDG-PET imaging to accurately predict Alzheimer’s-type tau pathology across spectrums of aging and dementia, providing a novel framework to estimate tau burden non-invasively.

## Introduction

The trans-modal prediction of Alzheimer’s disease (AD)-type tau across aging and AD and related disorders (ADRD) has become a major focus of research since the advent of positron emission tomography (PET) radioligands sensitive to this proteinopathy^1^. This effort is motivated by both scientific and practical impetuses. Scientifically, linking imaging modalities that capture distinct biological processes may clarify the mechanisms driving AD-type tau pathology and its downstream neurodegenerative effects. Practically, accurate prediction of this pathology may offer a cost-effective approach to enhance the efficiency of biological staging, prognostication, and longitudinal tau assessment.

Most trans-modal imaging models of AD-type tau propagation and neurodegeneration have relied on network-based frameworks, commonly referred to as the “prion-like” spreading hypothesis^2^. This hypothesis posits that functional and structural brain networks provide conduits through which pathogenic proteins propagate. In this framework, regions that are strongly connected to areas of high pathology (“epicenters”) are more likely to accumulate pathology, whereas weakly connected regions are less vulnerable. Ample empirical evidence supports this model in AD^3–7^, including its atypical variants^8–10^, as well as in primary tauopathies^6,11^, and neurodegenerative processes in non-AD syndromes, such as frontotemporal dementia (FTD)^12–14^. However, these studies are most often designed to simulate tau propagation dynamics at the region-level and typically not intended to be applied at the participant-level. Moreover, most of these investigations have relied on functional resting-state MRI, which, though valuable for probing brain physiology, is a noisy measure that constrains predictive accuracy and reliability at the participant-level^15^. It has also been suggested that the prion-like spreading hypothesis, while informative, may provide only a partial mechanistic account of tau propagation and neurodegeneration^16^.

Emerging computational models of brain function may offer additional insight into disease mechanisms and potentially improve predictive accuracy and clinical applicability^17^. It has been proposed that the brain’s functional architecture is structured along a hierarchy of large-scale gradients, which are continuous, low-dimensional axes that capture dominant patterns of spatial variation in brain function^18^. These gradients align with multiple biological features, including gene expression profiles^19,20^, neurotransmitter systems^21^, and metabolic regulation^22^. Importantly, large-scale gradients dynamically coordinate cognition^17,23,24^ and map onto the anatomical systems that support specific mental functions that are selectively degenerated across dementia syndromes^25,26^. Therefore, selective vulnerability to age-related proteinopathies such as AD-type tau may be embedded within these gradients^25,27,28^. We have previously demonstrated^25^ that a small set of gradients derived from fluorodeoxyglucose (FDG)-PET could map most common dementia syndromes within a global functional state space, with subspaces within this latent projection corresponding to the anatomical systems targeted in these disorders. Subsequent gradient-based approaches have since been applied in AD, helping to explain inter-individual variability in AD-type tau deposition and clinical symptoms^27,29^.

**Fig. 1:**
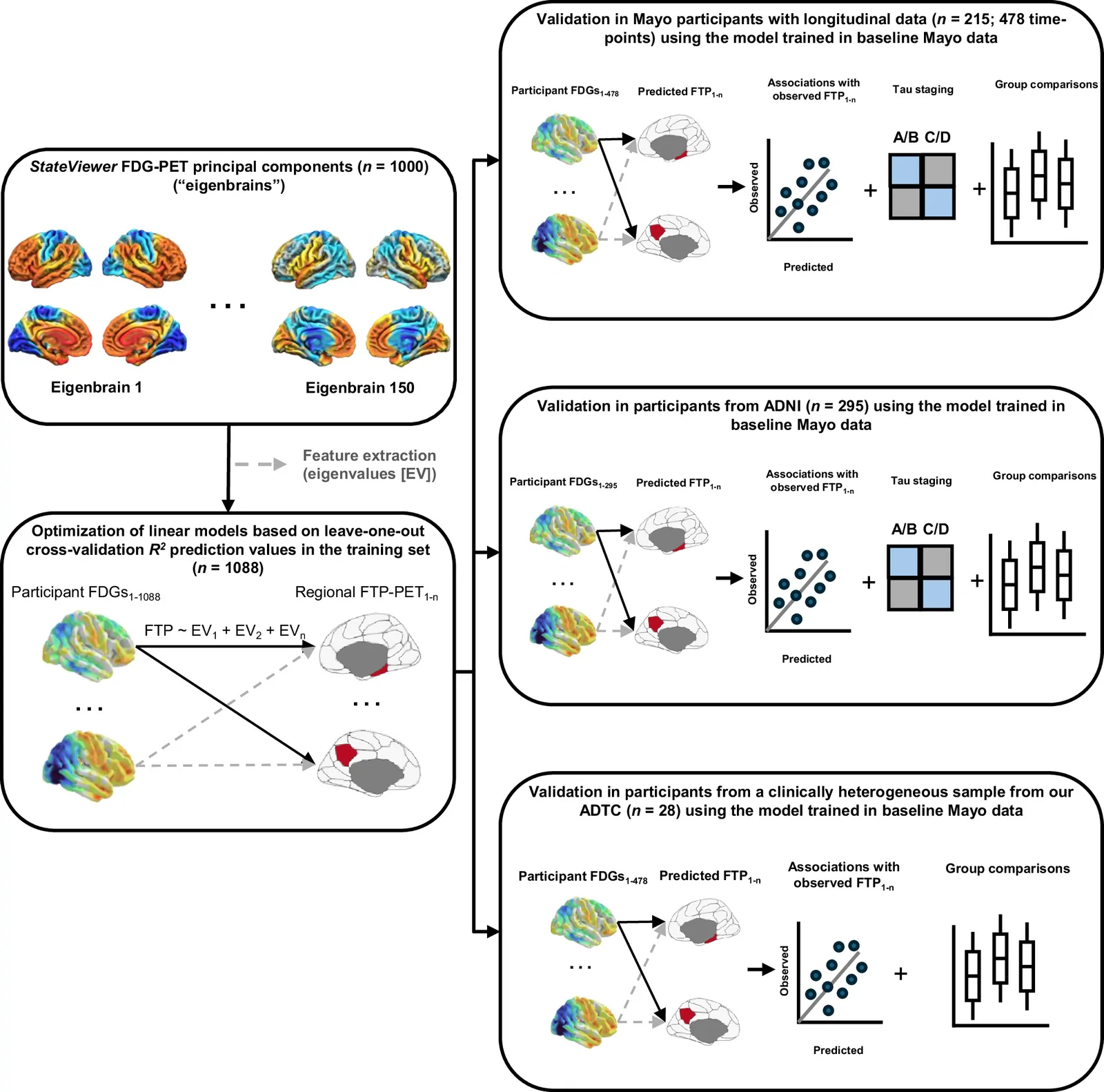
Analytic workflow of the FDG-PET AD-type tau prediction model. The approach involved the extraction of eigenvalues for the Mayo research cohort based on an independent latent space model (*StateViewer*). These eigenvalues were then used to predict FTP-PET across the Schaefer-400 atlas in the Mayo research cohort, and the best model was selected based on the optimal median leave-one-out cross-validation *R*^2^ predicted value. Models for the CenTauR atlas used the number of eigenbrains selected in the Schaefer-400 model. Validation was achieved via out-of-sample predictions of FTP-PET in participants from the Mayo research cohort with longitudinal data, ADNI participants, and ADTC patients using their corresponding *StateViewer*-derived eigenvalues and coefficients weights from the model trained in the Mayo research cohort. AD Alzheimer’s disease, ADTC AD treatment clinic, ADNI AD Neuroimaging Initiative, EV eigenvalue, FDG-PET Fluorodeoxyglucose positron emission tomography, FTP Flortaucipir.

In this study, we developed a scalable and interpretable model of AD-type tau prediction based on FDG-PET. In this study, we developed a scalable and interpretable model of AD-type tau prediction based on FDG-PET. We selected FDG-PET as our primary imaging modality because it is currently utilized in clinical practice for the evaluation of degenerative cognitive disorders to clarify differential diagnosis, index disease severity, and track progression^30,31^. Our model builds on *StateViewer*^32^, a clinical decision support system for the classification of degenerative dementia syndromes. *StateViewer* is derived from a principal component decomposition of 1000 randomly sampled FDG-PET scans and has demonstrated high classification accuracy across aging and nine neurodegenerative dementia syndromes (*n* = 3671), including robust out-of-sample performance. Here, we leveraged this existing framework to train our FDG-PET model of AD-type tau prediction in a large database of participants drawn from three Mayo Clinic research programs spanning the aging, AD, dementia with Lewy bodies (DLB), and FTD spectra (referred to as the “Mayo research cohort”) with Flortaucipir (FTP)-PET. The model is best framed as a population-level regression framework that leverages shared variance across a large cohort to estimate participant-level spatial distributions of FTP-PET burden. We validated the model in (1) participants from a Mayo Clinic research cohort with available longitudinal FDG-PET and FTP-PET data, (2) an external research dataset from the Alzheimer’s Disease Neuroimaging Initiative (ADNI)^33^, and (3) a clinically heterogeneous cohort of patients drawn from the Mayo Alzheimer’s Disease Treatment Clinic (ADTC)^34^, including a subset with available research FTP-PET. Figure 1 provides an overview of the analytic workflow.

In addition, we evaluated model performance across several clinically relevant tasks: (1) prediction of tau staging according to the Alzheimer’s Association’s revised criteria for the diagnosis and staging of AD^35^ in the training and validation cohorts; (2) prediction of tau stages in clinical syndromes with a high likelihood of co-pathologies (i.e., TDP-43 in amnestic mild cognitive impairment [aMCI] and amnestic AD [aAD])^36–38^ or that can result from either primary AD or non-AD pathologies (e.g., TDP-43 or tauopathies in behavioral variant FTD [bvFTD] or corticobasal syndrome [CBS])^39–42^. and (3) generation of biologically plausible tau patterns in clinically heterogeneous patients from the Mayo ADTC regardless of FTP-PET availability.

## Results

### Characterization of study cohorts

Study cohorts are described in Table 1. The Mayo research cohort (*n* = 1088) was used as the training set and included participants from research programs at Mayo Clinic Rochester (Mayo Clinic AD Research Center [ADRC], Mayo Clinic Study of Aging [MCSA], Neurodegeneration Research Group [NRG]) with amyloid-, FTP-, and FDG-PET. This cohort covered the AD, FTD, and DLB spectrum, in addition to aMCI, non-amnestic MCI (naMCI), and cognitively unimpaired (CU) participants. Overall, the cohort recapitulates known phenotype-specific features, for instance, a younger age in atypical AD phenotypes (dysexecutive AD [AD], logopenic variant primary progressive aphasia [lvPPA], posterior cortical atrophy [PCA]) and bvFTD, a male predominance in DLB, and a higher proportion of *APOE4* carriership in amnestic AD phenotypes (e.g., aMCI and amnestic AD [aAD]). A subset of the Mayo research cohort with longitudinal concurrent FTP-PET and FDG-PET was used as a validation cohort (*n* = 215, 478 time points total).

**Table 1:** Characterization of the Mayo Clinic research, ADNI, and ADTC cohorts

Group	*n*	Age	Female (%)	Education (years)	CDR-SB	*APOE4* (%)	A+ (%)	T+ (%)	Longitudinal PET (*n*)
Mayo research cohort									
CU A-T-	477	66 [57–73]	48	16 [14–17]	0 [0–0]	26	0	0	111
CU A + T–	144	75 [69–81]	50	15 [13–17]	0 [0–0]	40	100	0	30
CU A + T+	65	79 [73–85]	49	16 [14–16]	0 [0–0]	34	100	100	12
aMCI	29	75 [72–82]	31	16 [15–19]	1.5 [0.5–3.5]	69	100	76	10
naMCI	24	77 [72–81]	46	14 [12–16]	1 [0–2.6]	42	92	21	5
aAD	68	76 [71–80]	53	16 [14–18]	4 [1.5–6]	68	100	75	20
dAD	21	61 [57–65]	62	14 [14–16]	7 [5–9.5]	86	100	100	8
lvPPA	35	69 [61–74]	69	16 [13–16]	2.5 [1.5–13]	37	100	97	5
PCA	44	65 [61–72]	68	16 [14–16]	6.5 [3.3–7.5]	46	100	100	3
DLB	32	72 [67–80]	9	16 [12–17]	3.5 [2–5.6]	34	53	47	7
bvFTD	18	68 [61–74]	44	16 [13–18]	6.8 [2.6–10]	29	29	29	1
agPPA	41	70 [61–76]	61	16 [14–18]	1.8 [0.9–2.6]	32	24	17	1
PSP	51	71 [66–76]	41	17 [17–17]	0 [0–0]	24	14	14	0
SD	17	68 [62–73]	41	18 [16–18]	6.5 [4.4–8]	29	35	41	1
CBS	22	68 [64–73]	41	16 [12–16]	1 [0.3–2.8]	14	32	14	1
ADNI cohort									
CU	13	68 [66–72]	54	16 [14–18]	0 [0–0]	46	39	0	–
MCI	211	74 [68–79]	44	16 [14–18]	1 [0.5–2]	39	46	42	–
Dementia	71	77 [71–81]	42	16 [14–18]	4.5 [3.5–5.8]	65	76	70	–
ADTC cohort									
AD A+	138	73 [69–76]	59	16 [14–18]	2 [1.3–3.5]	80	100	–	–
Atypical AD A+	64	62 [59–68]	55	16 [14–18]	3 [1.5–5]	55	100	–	–
Non–AD A–	11	75 [72–78]	54	16 [14–18]	1 [0.5–3.5]	43	0	–	–
Non–AD A+	28	75 [72–78]	64	16 [13–16]	1 [0.6–2.1]	55	100	–	–
AD A+ co–path	23	79 [76–81]	44	16 [14–18]	3 [1.3–4.5]	70	100	–	–

Values are expressed as median with IQRs 25–75, percentage, or counts. *A* Amyloid, *aAD* amnestic Alzheimer’s disease, *ADNI* Alzheimer’s Disease Neuroimaging Initiative, *ADTC* Alzheimer’s Disease Treatment Clinic, *agPPA* Agrammatic primary progressive aphasia, *aMCI* amnestic mild cognitive impairment, *bvFTD* Behavioral variant frontotemporal dementia, *CBS* Corticobasal syndrome, *CDR-SB* clinical dementia rating sum of boxes, *CU* cognitively unimpaired, *dAD* dysexecutive Alzheimer’s disease, *DLB* Dementia with Lewy Bodies, *IQR* interquartile range, *lvPPA* Logopenic variant primary progressive aphasia, *MCI* Mild cognitive impairment, *naMCI* Non-amnestic mild cognitive impairment, *PET* Positron emission tomography, *PCA* posterior cortical atrophy, *PSP* Progressive supranuclear palsy, *SD* Semantic dementia, T +  Tau, *PET* positron emission tomography.

The ADNI cohort (*n* = 295) included CU, MCI, and Dementia participants with available amyloid-PET, FTP-PET, and FDG-PET imaging. This cohort was further classified according to amyloid and tau status for some of the analyses.

The ADTC cohort (*n* = 264) included amyloid-positive (A + ) patients with early symptomatic AD (“AD A + ”), early symptomatic AD with a high suspicion of co-pathology (e.g., α-synucleinopathy or TDP-43; “AD A+ co-path”), atypical AD variants (dAD, lvPPA, PCA, motor AD, or behavioral AD; “Atypical AD A + ”), and non-AD syndromes (e.g., normal pressure hydrocephalus, Parkinson’s disease, vascular dementia, frontotemporal dementia; “Non-AD”). The Non-AD group was further stratified by amyloid status into “Non-AD A + ” (i.e., non-AD symptom-driving etiology with positive amyloid testing) and “Non-AD A–” (i.e., non-AD symptom-driving etiology with negative amyloid testing). A subset of the ADTC cohort (*n* = 28) had research FTP-PET acquired as part of concomitant research programs (MCSA, ADRC; not included in the training cohort).

### FDG- and FTP-PET distribution across the ADRD spectrum in the Mayo research cohort

**Fig. 2:**
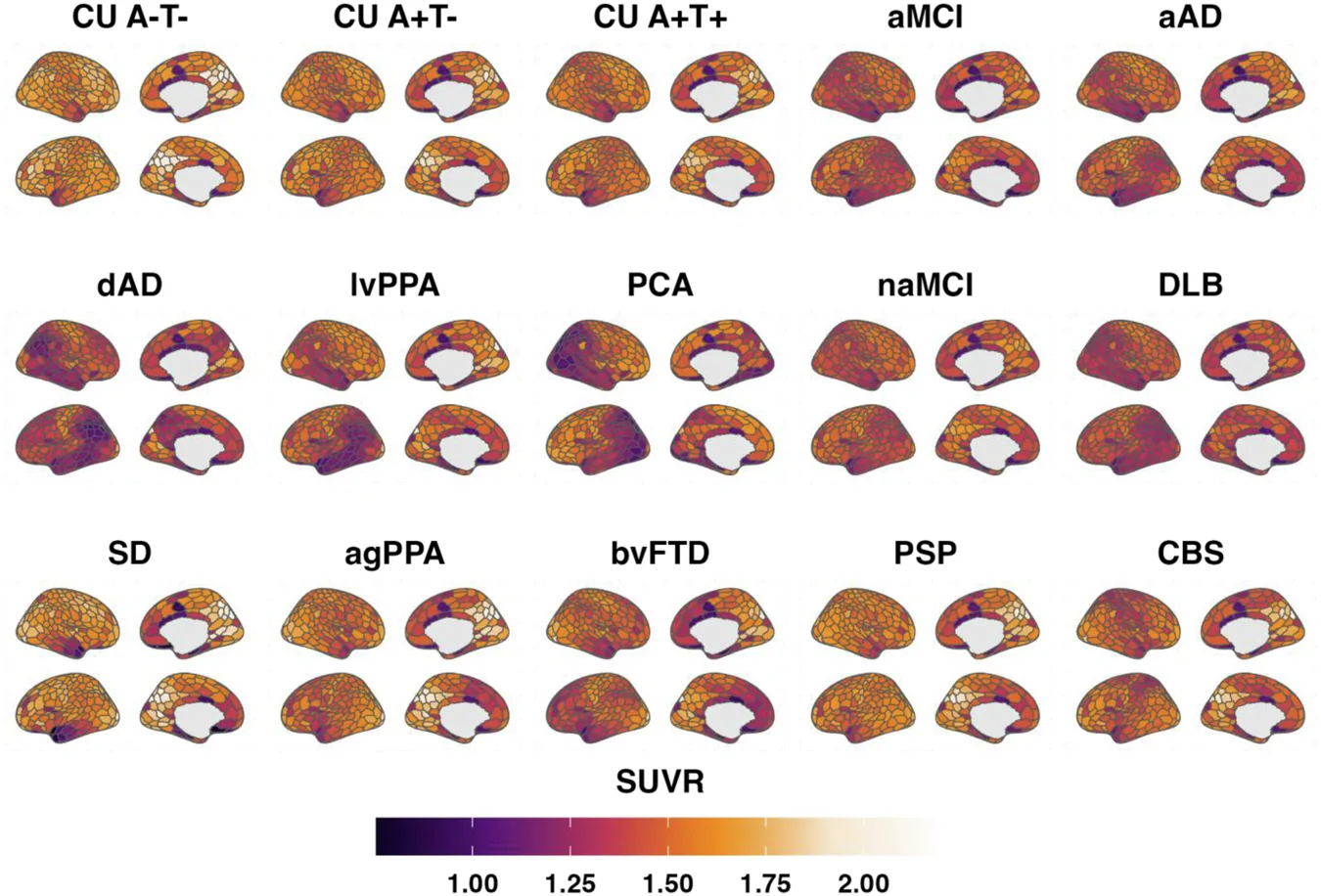
FDG-PET maps in the Mayo research cohort (training set). Values are organized so that more severe hypometabolism is reflected by darker colors. Brain renderings were generated using the *R* package ggseg. Source data are provided as a Source Data file. A +  Amyloid-positive, A- Amyloid-negative, aAD Amnestic Alzheimer’s disease, agPPA Agrammatic primary progressive aphasia, aMCI Amnestic mild cognitive impairment, bvFTD Behavioral variant frontotemporal dementia, CBS Corticobasal syndrome, CU Cognitively unimpaired, dAD Dysexecutive Alzheimer’s disease, DLB Dementia with Lewy Bodies, FDG-PET Fluorodeoxyglucose positron emission tomography, lvPPA Logopenic variant primary progressive aphasia, naMCI Non-amnestic MCI, PSP Progressive supranuclear palsy, SD Semantic dementia, SUVR Standard uptake value ratio, T + = Tau-positive, T- Tau-negative.

FDG-PET standard uptake value ratios (SUVR) distributions (Fig. 2) followed expected patterns, with CU groups reflecting natural patterns of age-related metabolism with higher SUVR values in metabolically demanding areas, including the precuneus, and lower values in regions with low metabolic demands such as the temporal pole and sensorimotor stip^22^. All symptomatic groups showed relatively diffuse hypometabolism throughout the cortex (i.e., lower SUVR values), with a predominance towards lateral temporal areas in aMCI and aAD, heteromodal cortex in dAD, left temporoparietal areas in lvPPA, occipito-temporal in PCA and DLB, temporal poles bilaterally in semantic dementia (SD), superior frontal and temporal areas in agrammatic PPA (agPPA), medial frontotemporal areas in bvFTD, medial frontal areas in progressive supranuclear palsy (PSP), and sensorimotor areas in CBS.

**Fig. 3:**
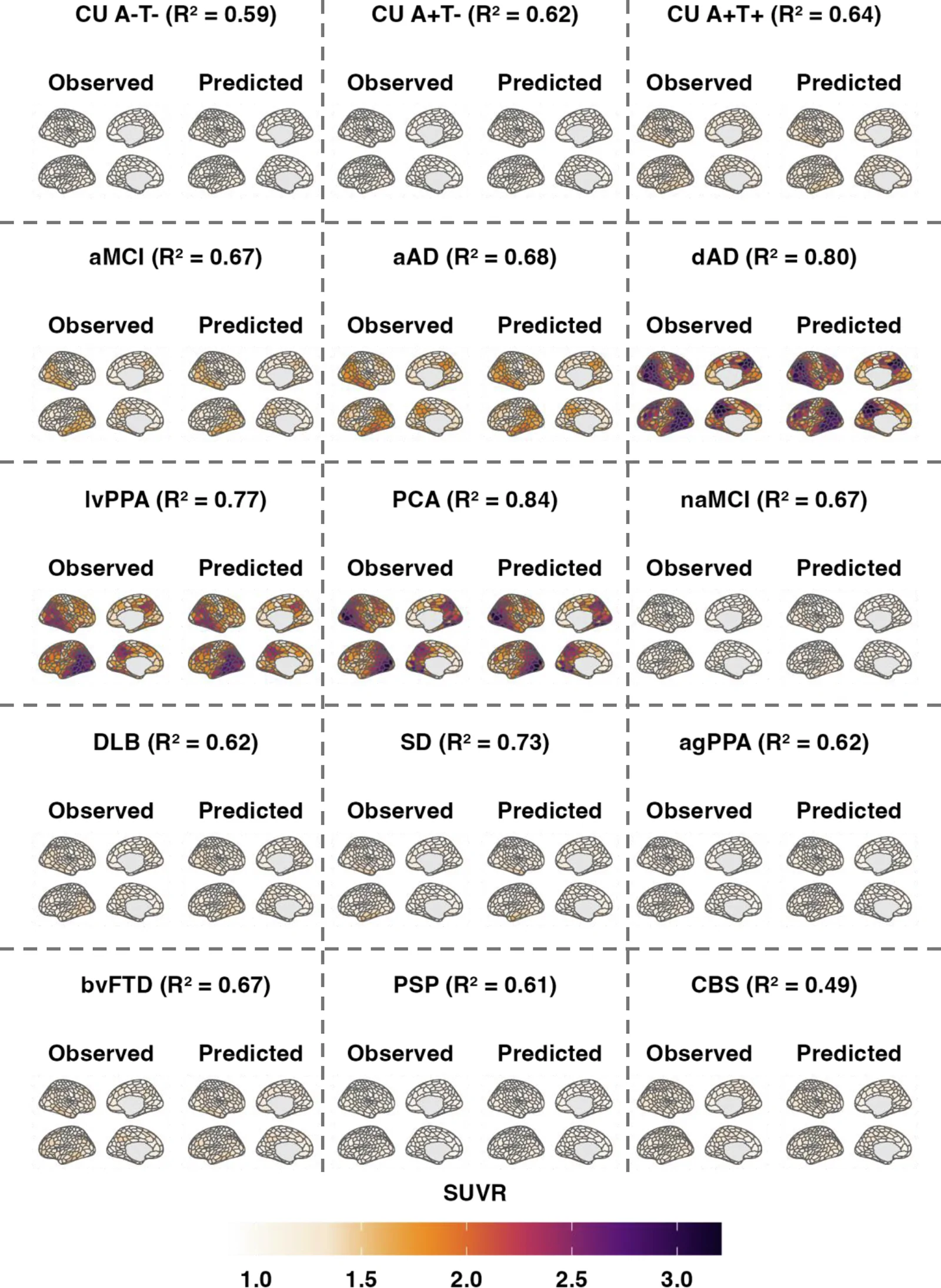
Observed and predicted FTP-PET maps from the FDG-PET eigenbrain model in the Mayo research cohort (training set). Predicted FTP-PET values across groups were derived from FDG-PET eigenvalues across the Mayo research cohort, using the optimal linear mixed-effect models based on 50 components (eigenbrains). *R*^2^ values for each observed-predicted pair indicate participant-level spatial correspondence between observed and predicted FTP-PET values, aggregated at the group level (median). Brain renderings were generated using the *R* package ggseg. Source data are provided as a Source Data file. A + = Amyloid-positive, A- Amyloid-negative, aAD Amnestic Alzheimer’s disease, agPPA Agrammatic primary progressive aphasia, aMCI Amnestic mild cognitive impairment, bvFTD Behavioral variant frontotemporal dementia, CBS Corticobasal syndrome, CU Cognitively unimpaired, dAD Dysexecutive Alzheimer’s disease, DLB Dementia with Lewy Bodies, FDG-PET Fluorodeoxyglucose positron emission tomography, FTP-PET Flortaucipir PET, lvPPA Logopenic variant primary progressive aphasia, naMCI Non-amnestic MCI, PSP Progressive supranuclear palsy, SD Semantic dementia, SUVR Standard uptake value ratio, T +  Tau-positive, T- Tau-negative.

The extent and distribution of group-level observed FTP-PET SUVR values (Fig. 3) also followed expected patterns, with greater uptake in the lateral temporal and medial parietal areas with more advanced disease stage from CU A + T+ to aMCI to aAD groups, whereas tau-negative CU groups had minimal uptake throughout the cortex. Atypical AD groups had the highest uptake that followed phenotype-specific patterns: the dAD group showed extensive uptake across the heteromodal cortex with relative sparing of the primary sensory and motor areas, the lvPPA group showed uptake in lateral temporoparietal areas with a leftward hemispheric predominance, and the PCA group showed prominent occipito-temporal uptake bilaterally. The naMCI and non-AD syndromes (DLB, SD, agPPA, bvFTD, PSP, CBS) showed minimal uptake throughout the cortex.

### Training of FDG-PET eigenbrain model in the Mayo research cohort

We projected the Mayo research cohort onto all principal components (eigenbrains; *n* = 150) from *StateViewer*^32^, an independent latent space model derived from 1000 randomly sampled FDG-PET images from Mayo Clinic. The first ten eigenbrains are shown in Supplementary Fig. 1. This yielded participant-level eigenvalues for each eigenbrain across the cohort. We then fit a series of linear regression models relating these eigenvalues to FDG-PET uptake in each region of the Schaefer-400 atlas. Models were iteratively tested with an increasing number of eigenbrains, and the optimal model was selected based on the highest overall leave-one-out *R*^*2*^_*pred*_ value. This was applied at the participant level, and we computed participant-level spatial *R*^2^ values across the atlas. Linear models applied across participants were benchmarked against five non-linear and regularized machine learning models (detailed in the Methods section), and a base model using age, sex, *APOE4* carriership, clinical diagnosis, and clinical dementia rating sum-of-boxes (CDR-SB) as predictors of FTP-PET instead of FDG-PET eigenbrains.

Additional regression models were performed across participants between eigenvalues and CenTauR values (mesial temporal tau, temporoparietal tau, temporal tau) to determine predicted tau stage based on the number of eigenbrains used in the final Schaefer-400 model. Clinically relevant case examples are presented, where observed versus predicted tau stages/burden were compared in aMCI, aAD, bvFTD, and CBS groups.

The best-performing model included 50 eigenbrains as predictors of regional FTP-PET uptake across participants, achieving median *R*^*2*^ = 0.71 [IQR 0.61 to 0.77], *R*^*2*^_*adj*_ = 0.69 [IQR 0.59 to 0.76], *R*^*2*^_*pred*_ = 0.65 [IQR 0.54 to 0.73], mean absolute error (MAE) = 0.09 [IQR 0.08 to 0.09], and mean squared error (MSE) = 0.01 [0.01 to 0.02] across the Schaefer-400 atlas. Participant-level spatial comparisons of observed and predicted FTP-PET values across the 400 regions of the atlas achieved a median value of *R*^*2*^ = 0.79 [0.73 to 0.84].

**Fig. 4:**
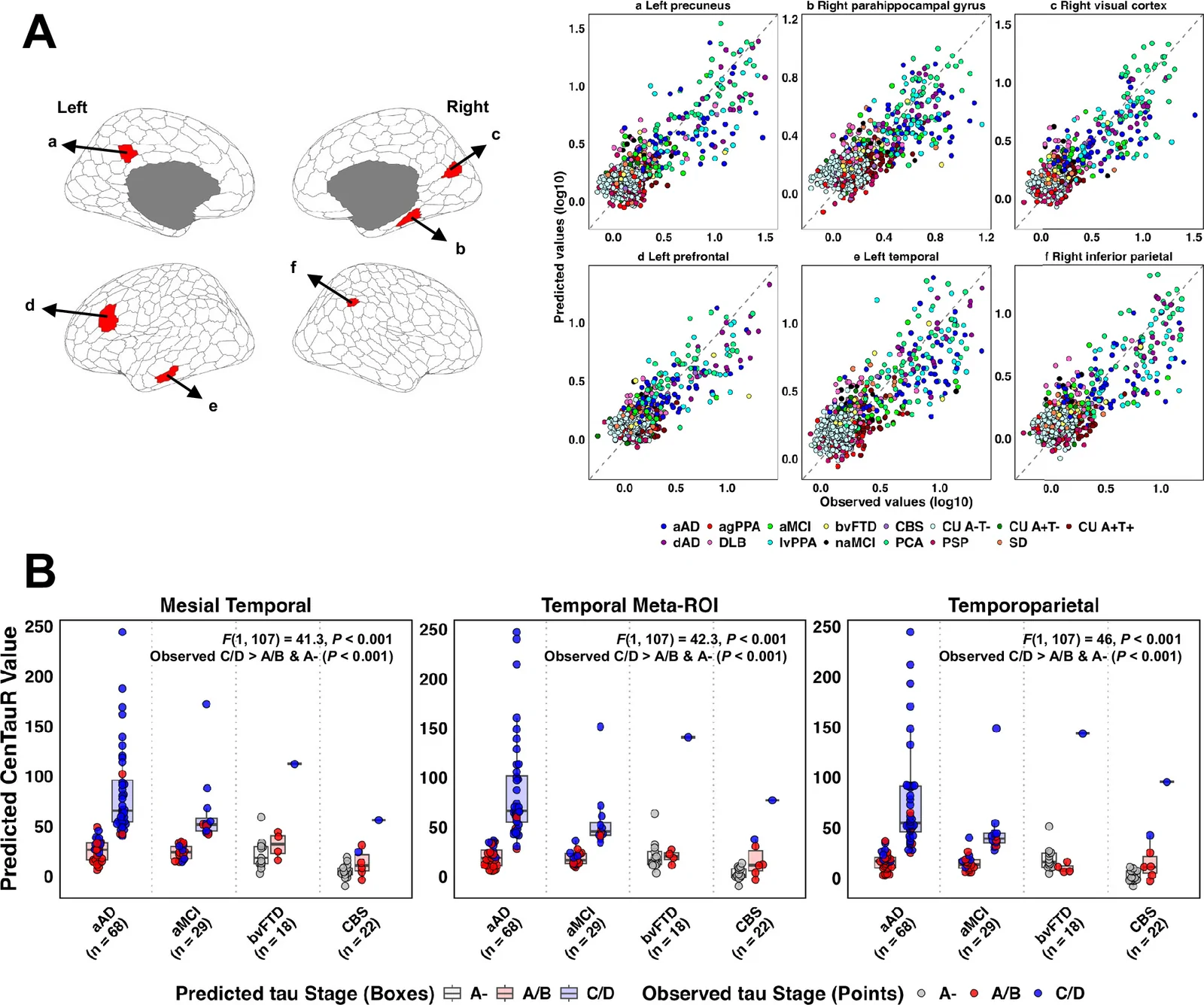
Comparisons between observed and predicted FTP-PET values in the Mayo research cohort (training set). **A** shows region examples where each dot represents a participant, color-coded by diagnosis. **B** shows CenTauR values in case examples of aAD, aMCI, bvFTD and CBS groups, color-coded by tau stage with observed tau stage identified by dots and predicted tau stage identified by boxes. Statistics in inset indicate comparisons of predicted CenTauR values across observed tau stages. Box plots show the median (center line), upper and lower quartiles (box bounds, 25 and 75th percentiles), and minimum to maximum values within 1.5× the interquartile range (whiskers). Brain renderings were generated using the Rpackage ggseg. Source data are provided as a Source Data file. A +  Amyloid-positive, A- Amyloid-negative, aAD Amnestic Alzheimer’s disease, agPPA Agrammatic primary progressive aphasia, aMCI Amnestic mild cognitive impairment, bvFTD Behavioral variant frontotemporal dementia, CBS Corticobasal syndrome, CU Cognitively unimpaired, dAD Dysexecutive Alzheimer’s disease, DLB Dementia with Lewy Bodies, FTP Flortaucipir, lvPPA Logopenic variant primary progressive aphasia, ROI Region of interest, PSP Progressive supranuclear palsy, SD Semantic dementia, SUVR Standard uptake value ratio, T +  Tau-positive, T- Tau-negative.

Figure 3 depicts predicted values from the model, which show relatively similar patterns across groups compared to observed FTP-PET uptake values. Figure 4 (panel A) shows observed and predicted FTP-PET values in six region examples. Supplementary Fig. 2 shows performance metrics across Yeo’s networks^43^, and Supplementary Fig. 3 shows three-dimensional brain renderings of *R*^2^ values for each group, with markedly higher values in groups with a wider FTP-PET dynamic range (e.g., dAD, PCA) than groups with minimal FTP-PET signal (e.g., CU groups, SD, PSP). Supplementary Fig. 4 shows Bland-Altman plots across all diagnostic groups. While the overall mean predicted values generally fall within the 95% limits of agreement of the observed values, high-tau groups (i.e., aAD, dAD, and lvPPA) exhibit a slight degree of proportional bias, characterized by overestimation at lower mean values and underestimation at higher mean values.

Repeating the analyses accounting for age, sex, and *APOE4* status did not improve model performance, achieving median *R*^*2*^ = 0.71 [IQR 0.62 to 0.78], MAE = 0.09 [IQR 0.08 to 0.09], and MSE = 0.1 [IQR 0.01 to 0.2]. Therefore, further analyses were performed without covariates. However, in order to explicitly assess performance across sexes, *R*^2^ values from the main model were compared between males and females across the Schaefer-400 atlas. This yielded higher values in females (*R*^*2*^ = 0.75 [IQR 0.66–0.81]) compared to males (*R*^*2*^ = 0.61 [IQR 0.5–0.70]).

Comparisons with alternative non-linear and regularized models revealed lower performance, with *R*^2^ values ranging from 0.59 to 0.65. The base model including age, sex, *APOE* carriership, diagnosis, and CDR-SB also achieved lower performance, with an *R*^2^ value of 0.51. Full performance metrics can be found in Supplementary Table 1.

The regression model between FDG-PET eigenvalues and CenTauR values yielded *R*^*2*^ = 0.59 [IQR 0.54 to 0.60], MAE = 0.23 [IQR 0.22 to 0.25], and MSE = 0.11 [IQR 0.10 to 0.12] across the mesial temporal, temporoparietal, and temporal meta-ROI regions across participants. A confusion matrix excluding A- participants (as this stage is determined by amyloid status only) achieved an overall accuracy of 88%. In total, 290 cells were correctly labeled as A/B, 134 cells were correctly labeled as C/D, 20 cells were incorrectly labeled as A/B, and 36 cells were incorrectly labeled as C/D. Re-running the model including A- individuals achieved an overall accuracy of 95%. Full performance metrics are shown in Supplementary Table 2.

The case examples in aMCI, aAD, bvFTD, and CBS groups showed an overall accuracy of 82% when excluding A- individuals, with 42 cells correctly labeled as A/B, 47 cells correctly labeled as C/D, 7 cells incorrectly labeled as A/B, and 13 cells incorrectly labeled as C/D. Including A- individuals yielded an overall accuracy of 85%. Full performance metrics are shown in Supplementary Table 3.

Group-wise comparisons of predicted CenTaur values achieved significant results for the mesial temporal lobe *F*(1, 107) = 41.3, *P* < 0.001, temporoparietal area *F*(1, 107) = 42.3, *P* < 0.001, and temporal meta-ROI *F*(1, 107) = 46, *P* < 0.001. Tukey post-hoc analyses indicated higher predicted values in individuals with an observed C/D stage compared to observed A- and A/B stages (*P* < 0.001 in all cases), and higher values in A/B stage compared to A- (*P* < 0.05). Results from this analysis are shown in Fig. 4 (panel B). Supplementary Fig. 5 visually depicts predicted CenTaur values across all groups, stratified by predicted tau stage.

### Validation in the longitudinal Mayo research cohort

The first set of validation analyses involved the participants from the Mayo research cohort with longitudinal FTP-PET and FDG-PET data. Predicted Schaefer-400 and CenTauR values were extracted using the model trained on baseline data and *StateViewer*-derived eigenvalues at each time point for each participant. Mixed-effects models between observed and predicted values across the Schaefer-400 atlas yielded a marginal *R*^*2*^ = 0.51 [IQR 0.43 to 0.67], MAE = 0.03 [IQR 0.03 to 0.03], and MSE = 0.001 [IQR 0.001 to 0.002] across participants. Participant-level spatial comparisons of observed and predicted FTP-PET values across visits achieved a median value of *R*^*2*^ = 0.78 [0.71 to 0.83].

**Fig. 5:**
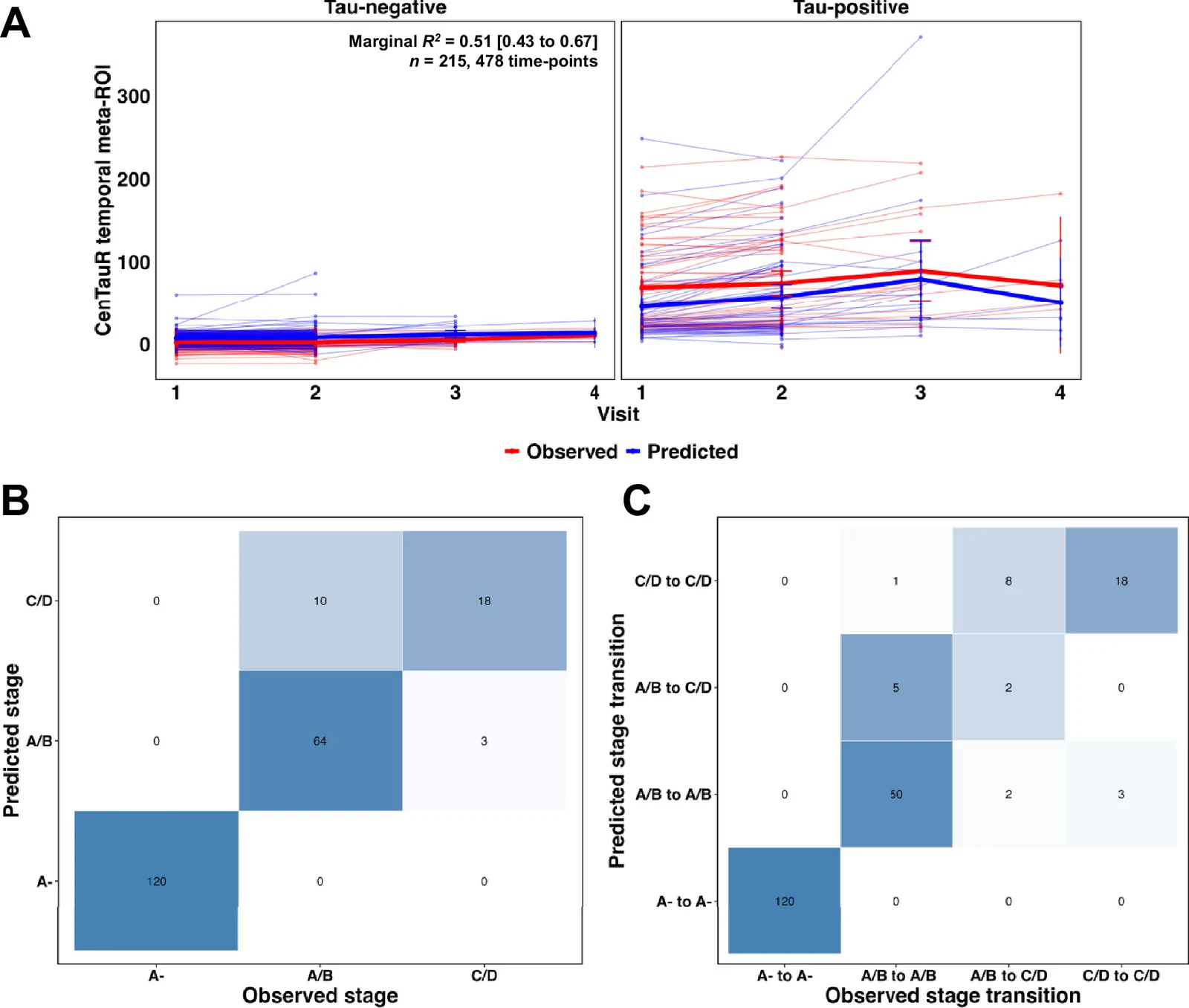
Results in the subset of the Mayo research cohort with longitudinal data (validation set). **A** shows observed and predicted CenTauR temporal meta-ROI values across the whole sample, stratified by observed tau status. Data points represent individual trajectories, with bold lines showing group means. Error bars denote 95% confidence intervals of the mean, calculated assuming a normal distribution. **B** shows the confusion matrix of the agreement between observed and predicted tau stages at baseline. **C** shows the confusion matrix of the agreement between observed and predicted tau stage transitions over time. Source data are provided as a Source Data file. ROI Region of interest.

The regression model between FDG-PET eigenvalues and CenTauR values yielded a marginal *R*^*2*^ = 0.57 [0.49 to 0.58], MAE = 0.06 [0.06 to 0.07], and MSE = 0.01 [0.01 to 0.01] across participants. A confusion matrix at baseline that excludes A- participants achieved an overall accuracy of 86%, with 64 cells correctly labeled as A/B, 18 cells correctly labeled as C/D, 3 cells incorrectly labeled as A/B, and 10 cells incorrectly labeled as C/D. Including A- individuals resulted in an accuracy of 94%. A confusion matrix comparing observed and predicted tau stage transitions between the first and last FTP-PET scans for each participant while excluding A-participants achieved an overall accuracy of 80%. A confusion matrix including A- participants achieved an accuracy of 91%. Longitudinal observed versus predicted CenTauR temporal meta-ROI and confusion matrices are shown in Fig. 5. Performance metrics for all confusion matrices can be found in Supplementary Table 4.

Analyses were repeated using a baseline training model without participants with longitudinal data to assess whether overlap between the training and validation set influences performance. Performance of mixed-effect linear models across the Schaefer-400 atlas across participants yielded lower performance, with a marginal *R*^*2*^ = 0.39 [IQR 0.33–0.46], MAE = 0.03 [IQR 0.03–0.03], and MSE = 0.002 [IQR 0.001 to 0.002]. Performance of mixed-effect linear models across the CenTauR regions yielded marginal *R*^*2*^ = ~ 0.33–0.47, MAE = ~0.06–0.07, and MSE = ~0.007–0.009. However, this had no impact on tau staging classification results, which remained identical to the original model.

Finally, we evaluated whether baseline prediction residuals between predicted and observed FTP-PET in the CenTauR temporal meta-ROI carry prognostic value by assessing their relationship with change (Δ) in CDR-SB and the change (Δ) in CenTauR temporal meta-ROI between the baseline visit and the final follow-up visit for each participant. This yielded a small yet significant relationship between baseline residuals and Δ CDR-SB, *ρ* = 0.231, *P* = < 0.001 and between baseline residuals and Δ temporal meta-ROI, *ρ* = 0.125, *P* = 0.04. This indicates that individuals for whom FTP-PET burden was underestimated at baseline tended to clinically worsen and accumulate more tau over time. These relationships are visually depicted in Supplementary Fig. 6.

### Validation in the ADNI cohort

**Fig. 6:**
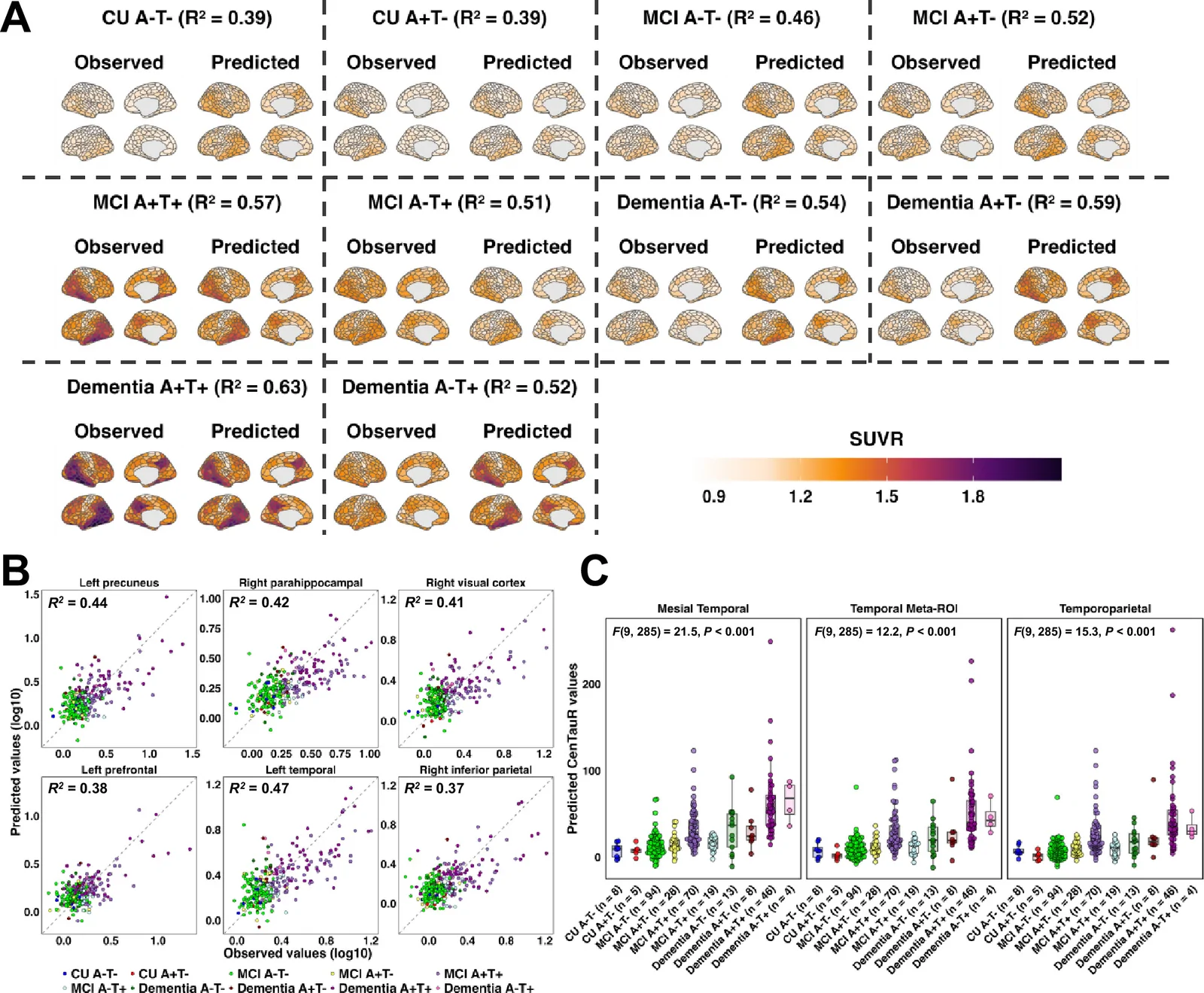
Results in the ADNI cohort (validation set). **A** shows renderings of predicted FTP-PET values across groups. *R*^2^ values for each observed-predicted pair indicate participant-level spatial correspondence between observed and predicted FTP-PET values, aggregated at the group-level (median). **B** shows region examples where each dot represents a participant, color-coded by diagnosis. **C** shows group comparisons of predicted CenTauR values across groups. Box plots show the median (center line), upper and lower quartiles (box bounds, 25 and 75th percentiles), and minimum to maximum values within 1.5× the interquartile range (whiskers). Brain renderings were generated using the *R* package ggseg. Source data are provided as a Source Data file. A+ Amyloid-positive, A- Amyloid-negative, AD Alzheimer’s disease, FTP Flortaucipir, MCI Mild cognitive impairment, ROI Region of interest, T+ Tau-positive, T- Tau-negative, SUVR Standard uptake value ratio.

The second set of validation analyses involved out-of-sample prediction in the ADNI cohort. Predicted Schaefer-400 and CenTauR values were extracted using the model trained in the Mayo research cohort and *StateViewer*-derived eigenvalues in ADNI participants. Mixed-effect models between observed and predicted FTP-PET across the Schaefer-400 atlas achieved *R*^*2*^ = 0.33 [IQR 0.21 to 0.41], MAE = 0.11 [IQR 0.10 to 0.12], and MSE = 0.02 [IQR 0.02 to 0.03] across participants. Individual spatial *R*^2^ across the cohort achieved a median value of *R*^*2*^ = 0.72. Figure 6 shows renderings of predicted FTP-PET values (panel A), group-wise comparisons (panel B), and observed and predicted FTP-PET values in six region examples (panel C).

The regression model between FDG-PET eigenvalues and CenTauR values yielded *R*^*2*^ = 0.44 [IQR 0.42 to 0.44], MAE = 0.27 [IQR 0.26 to 0.30], and MSE = 0.12 [IQR 0.11 to 0.15]. Comparisons between observed and predicted tau burden with combined stages A/B and C/D without A- individuals achieved an accuracy of 79%, with 82 cells correctly labeled as A/B, 41 cells correctly labeled as C/D, 12 cells incorrectly labeled as A/B, and 22 cells incorrectly labeled as C/D. Analyses including A- individuals achieved an accuracy of 89%. Performance metrics for all analyses can be found in Supplementary Table 5.

Comparisons of predicted CenTauR regions across clinically- and biomarker-defined groups showed a significant effect for the mesial temporal area, *F*(9, 285) = 21.5, *P* < 0.001, temporoparietal area, *F*(9, 285) = 12.2, *P* < 0.001, and temporal meta-ROI, *F*(9, 285) = 15.3, *P* < 0.001. Post-hoc analyses revealed that, across all regions, the Dementia A + T+ group had higher values than the CU A-T-, CU A + T-, Dementia A-T-, and all MCI groups, and that the MCI A + T+ group had higher values than the MCI A-T- and MCI A + T- groups. In addition, post-hoc analyses for the mesial temporal lobe revealed higher values in the Dementia A + T+ group compared to the Dementia A + T- group, and higher values in the Dementia A-T+ group compared to the CU A-T-, CU A + T-, MCI A-T-, MCI A-T + , and MCI A + T- groups. Figure 6 shows predicted FTP-PET values across groups.

### Validation in the ADTC cohort

**Fig. 7:**
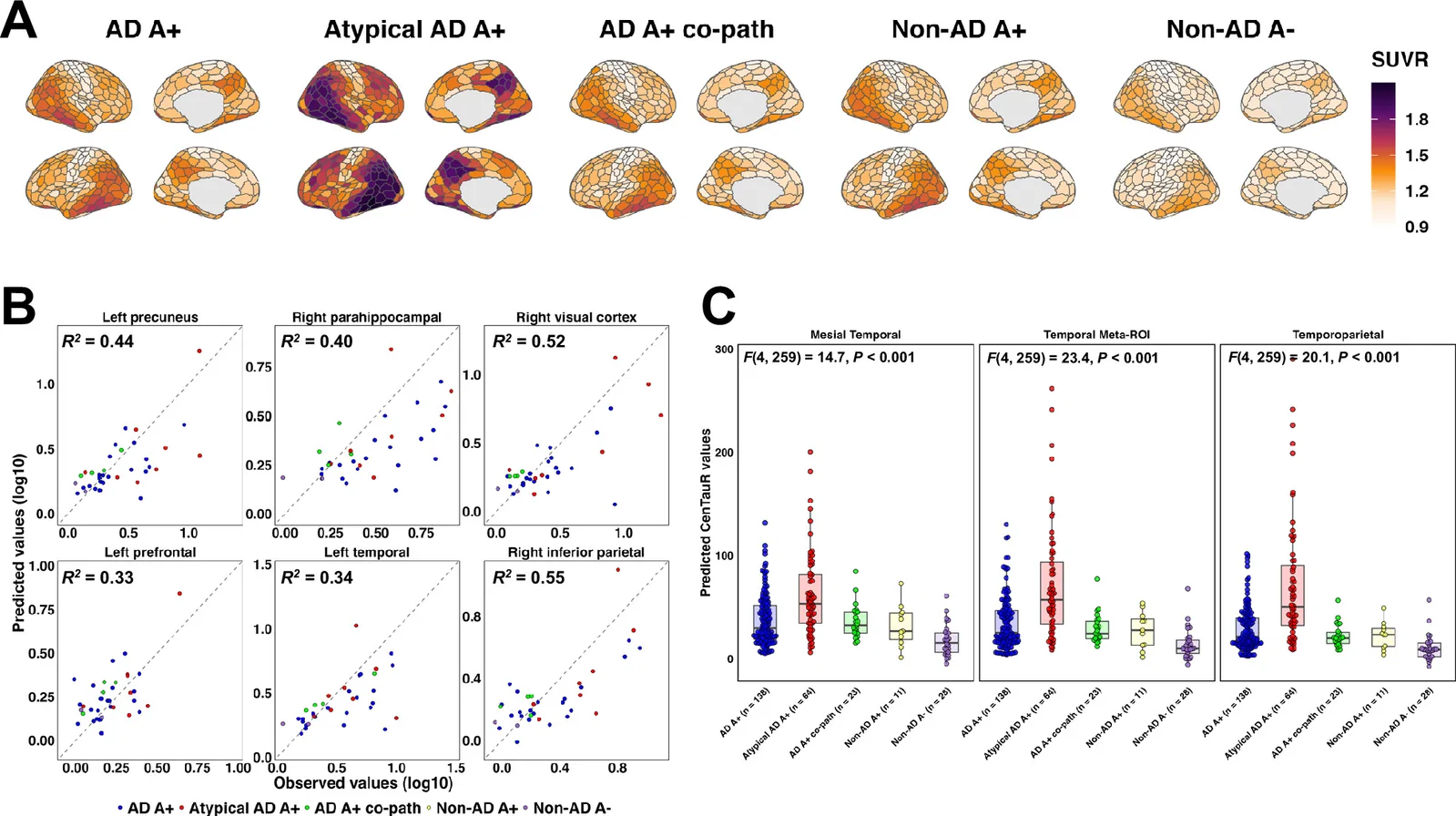
Results in the ADTC cohort (validation set). **A** shows renderings of predicted FTP-PET values across groups. **B** shows region examples where each dot represents a participant, color-coded by diagnosis. **C** shows group comparisons of predicted CenTauR values across groups. Box plots show the median (center line), upper and lower quartiles (box bounds, 25th and 75th percentiles), and minimum to maximum values within 1.5× the interquartile range (whiskers). Brain renderings were generated using the *R* package ggseg. Source data are provided as a Source Data file. A +  Amyloid-positive, A- Amyloid-negative, AD Alzheimer’s disease, FTP Flortaucipir, ROI Region of interest, SUVR Standard uptake value ratio.

The third set of validation analyses involved out-of-sample prediction in ADTC patients with available FTP-PET (*n* = 28). Predicted Schaefer-400 and CenTauR values were extracted using the model trained in the Mayo research cohort and *StateViewer*-derived eigenvalues in ADTC participants. Mixed-effect models between observed and predicted FTP-PET across the Schaefer-400 atlas achieved *R*^*2*^ = 0.41 [IQR 0.27 to 0.52], MAE = 0.12 [IQR 0.09 to 0.14], and MSE = 0.02 [IQR 0.01 to 0.04] across participants. Individual spatial *R*^2^ across the cohort achieved a median value of *R*^*2*^ = 0.80. Figure 7 shows brain renderings of predicted FTP-PET values (panel A), group-wise comparisons (panel B), and observed and predicted FTP-PET values in six region examples (panel C).

The regression model between FDG-PET eigenvalues and CenTauR values yielded *R*^*2*^ = 0.31 [IQR 0.23 to 0.42], MAE = 0.30 [IQR 0.27 to 0.30], and MSE = 0.16 [IQR 0.13 to 0.17] across participants. Comparisons of predicted CenTauR regions across clinical groups regardless of FTP-PET availability showed a significant effect for the mesial temporal area, *F*(4, 259) = 14.7, *P* < 0.001, temporoparietal area, *F*(4, 259) = 23.4, *P* < 0.001, and the temporal meta-ROI area, *F*(4, 259) = 20.1, *P* < 0.001. Post-hoc analyses revealed that, across all regions, the Atypical AD A+ group had higher values than all other groups. In addition, the AD A+ group had higher values than the Non-AD A- group for the mesial temporal lobe and temporal meta-ROI regions. Figure 7 shows predicted FTP-PET values across groups.

## Discussion

The trans-modal prediction of AD-type tau across the ADRD spectrum is critical for advancing our understanding of this pathology and its relationship to large-scale functional dynamics and neurodegeneration. In this study, we developed a highly interpretable model of FTP-PET prediction based on FDG-PET imaging and a population-based regression framework, implemented within *StateViewer*^32^, a clinical decision support system for dementia syndrome classification based on a computational model of brain function. The model demonstrated high accuracy in the training set in predicting the extent and distribution of FDG-PET as well as tau staging across the ADRD spectrum, including CU individuals with and without positive AD biomarkers, clinical AD phenotypes, and non-AD syndromes spanning the DLB and FTLD spectra. Moderate-to-high performance in longitudinal data and independent datasets from research and clinical practice further highlight its potential for generalization and scalability.

Predictions from the FDG-PET eigenbrain model closely matched observed FTP-PET when applied across and within participants. It accurately captured high FTP-PET burden in atypical AD phenotypes, moderate levels in aMCI and aAD, and lower levels in CU, naMCI, and non-AD groups. The prediction of low FTP-PET in non-AD primary tauopathies (e.g., PSP, CBS) was expected, given the lower binding sensitivity of existing FTP-PET tracers to non-AD tau^44,45^. This also supports the model’s specificity to AD-type tau in that predictions are driven by specific patterns of whole-brain metabolism rather than simply translating regional FDG-PET signal into spatially equivalent FTP-PET patterns.

This work carries several practical implications. Our FDG-PET–based model can approximate FTP-PET burden across the ADRD spectrum using FDG-PET imaging as a single input, consistent with prior work from our group^46^. Generalization to longitudinal data and independent ADTC and ADNI cohorts suggests potential scalability to unseen data. Moreover, our model achieved high accuracy (78–95%) in predicting tau staging cross-sectionally and longitudinally both within the training and validation cohorts. This highlights its potential ability to support biological staging under revised diagnostic criteria for AD^35^, enabling stratification of participants by low versus high tau burden. To this point, clinically relevant case examples in amnestic phenotypes of AD (aMCI/aAD) and syndromes that can be caused either by AD or non-AD etiologies (bvFTD, CBS) demonstrated that the model accurately predicted low tau burden in those with observed low tau (A/B stages). Similar results were found in the ADNI cohort by comparing predicted tau values across CU, MCI, and Dementia patients according to observed biomarker status. This suggests that our model could potentially (1) identify “off-diagonal” symptomatic individuals with possible symptom-driving co-pathologies^35,37^, and (2) identify rare AD phenotypes that are traditionally caused by non-AD etiologies. FDG-PET could also be used to infer longitudinal FTP-PET burden when the latter is unavailable or onerous. Overall, our model provides a highly scalable surrogate that maximizes the clinical and research yield of FDG-PET, and supports the role of this imaging modality as a close proxy of AD-type tau accumulation^47–50^, related neurodegeneration, and clinical symptoms^25,30,32^,

Our findings are also relevant to broader conceptual frameworks of neurodegeneration. The FDG-PET model is grounded in the principle of shared vulnerability across large-scale brain systems^18,25^. In this view, functional gradients that support specific cognitive domains shape the topography of neurodegeneration across dementia syndromes^25,28^. Such vulnerability likely arises from commonalities and differences in genetic expression, metabolic demands, and cellular properties within and across gradients^18–20,51–54^. Importantly, these results do not contradict the prion-like spreading hypothesis in non-prion diseases. Instead, gradient-based and epicenter-based frameworks evaluate similar questions at different scales, and converge on the notion that large-scale functional systems are central to disease biology and may even act not only as mere conduits but also drivers of proteinopathies in later life, an aspect often underappreciated in molecular pathology models^25,55–57^. From this perspective, tau and other pathogenic drivers may accumulate preferentially along syndrome-specific gradients rather than arising from isolated focal sites, rendering the gradients themselves “disease epicenters.” Our framework helps explain why some large-scale systems are disproportionately vulnerable to AD-type tau while others remain relatively resistant. This is supported by the model’s capacity to predict an absence of FTP-PET signal in dementia syndromes where tau is not the primary driver of neurodegeneration, even in the presence of significant FDG-PET abnormality. Future work linking molecular physiology, genetic expression profiles, and large-scale network organization will be essential to clarify mechanisms of pathologic AD-type tau genesis and propagation^1^.

It is also noteworthy that while our model explains a high proportion of variance in patterns of FTP-PET, additional factors may influence inter-individual variability. For instance, post-hoc analyses on baseline residuals and rates of change in CDR-SB and temporal FTP-PET support the notion that variance unexplained by our model carries biological meaning and prognostic value. A potential contributing factor is biological sex, as supported by distinct differences in the proportion of variance explained between females and males in our post-hoc stratified analyses. This aligns with extant literature demonstrating sex-specific effects on trajectories of AD-type tau accumulation that disfavor women^58^. In addition, the model showed a slight degree of proportional bias in high-tau groups (i.e., dAD, PCA, lvPPA). However, this type of bias for extreme values was expected given the linear assumptions of the model. Future work exploring such factors is needed not only to improve the accuracy of data-driven tools, but also to clarify the mechanistic underpinnings of AD-type tau progression.

These findings should be interpreted in light of several limitations. First, the model has not yet been evaluated in non-degenerative conditions that can clinically mimic neurodegenerative syndromes, such as autoimmune disorders, cerebrospinal fluid dynamics, and primary psychiatric disorders. Second, model training was limited to the Mayo research cohort, which is predominantly non-Hispanic white and highly educated. Although out-of-sample performance supports generalizability, further validation in racially, ethnically, and socioeconomically diverse populations is warranted. Third, while we used CenTauR based on prior work^59^, FTP-PET thresholds for biological stages are not well defined in the literature. In addition, there was evidence of class imbalance in tau stages, with a minimal proportion of cases presenting with intermediate tau stages (B/C). This warrants validation in future cohorts featuring a more evenly distributed representation of tau stages. Fourth, despite acceptable out-of-sample performance in both the ADNI and ADTC cohorts, further cross-center validation is needed. Fifth, the baseline data from the Mayo longitudinal validation cohort overlapped with the training cohort. Moreover, only a small number of individuals transitioned to a higher tau stage. While sensitivity analyses support robust generalization, further replication in completely independent longitudinal datasets is needed. Sixth, our model was only trained on FTP-PET imaging, and it remains to be tested whether it would perform similarly using different ligands with a broader dynamic range, or even recent ligands that may bind to primary tauopathies such as PI2620^60^. Seventh, it is important to note that the ADNI cohort is heavily pre-screened for AD-centric symptoms and biology, and is minimally representative of real-world clinical populations. While the inclusion of the ADTC cohort mitigates this to some degree, replication in larger, clinically heterogeneous cohorts is warranted. Finally, an important future direction of this work is the assessment of integrating FDG-PET markers and plasma biomarkers into a minimal, cost-effective clinical workflow for diagnostic purposes. While our standalone imaging model provides robust utility when fluid biomarkers are missing, combining spatial metabolic predictions with highly accessible plasma assays could further refine differential diagnosis in complex clinical settings^61^.

In summary, we developed a multimodal model of tau prediction based on a framework using FDG- and FTP-PET imaging and machine learning within a functional gradient paradigm. The model achieved high accuracy across the ADRD spectrum, shows strong potential for scalability and generalization, and may provide a practical tool to improve the efficiency of research and clinical efforts that rely on tau-PET staging.

## Methods

### Mayo clinic research cohort

Patients and/or their legal representative provided written consent for their data to be used for research purposes. This study met HIPAA guidelines and was approved by the Mayo Clinic Institutional Review Board. Research participants were financially compensated for their participation in the ancillary studies to this research.

The Mayo research cohort included 1088 participants enrolled through research programs at Mayo Clinic Rochester, including the ADRC, the MCSA, and the NRG. The cohort spanned the spectrum of normal aging, AD, DLB, and FTLD. AD phenotypes had to be A+ based on PET imaging and included: aMCI^62^, aAD^63^, dAD^64^, lvPPA^65^, and PCA^66^. FTD phenotypes included bvFTD^67^, agPPA, SD^65^, CBS^39^ and PSP^68^. naMCI participants did not have to be A+ to be included, as they often reflect the prodromal stage of non-AD disorders. CU participants covered the AD biomarker spectrum (A-T-, A + T-, A + T + )^35,69^. All participants had FDG-PET, amyloid-PET, and FTP-PET imaging acquired within a 6-month window. A subset of participants had at least one pair of follow-up FDG-PET and FTP-PET (*n* = 215, total of 478 time points) and was used as a validation cohort.

Clinical diagnoses were determined by dementia specialists using standardized protocols established by the National Institute on Aging–funded ADRC program^70^. This included a structured clinical interview with the participant and an informant, focused on core diagnostic features of the relevant syndromes described above, along with a neurological examination, review of medical records, and ancillary data (e.g., neuroimaging, neuropsychological testing), when appropriate.

### ADNI cohort

Participants from ADNI (adni.loni.usc.edu) were used as a second validation cohort (*n* = 295). This cohort included participants with available amyloid-PET, FTP-PET, and FDG-PET imaging, including participants labeled as CU (*n* = 13), MCI (*n* = 211), or dementia (*n* = 71). Participants were further classified according to biomarker status, with CU A-T- (*n* = 8), CU A + T- (*n* = 5), MCI A-T- (*n* = 94), MCI A + T- (*n* = 28), MCI A + T+ (*n* = 70), MCI A-T+ (*n* = 19), Dementia A-T- (*n* = 13), Dementia A + T- (*n* = 8), Dementia A + T+ (*n* = 46) and Dementia A-T+ (*n* = 4). Clinical diagnoses were assigned according to standardized protocols described in the ADNI methods^33^.

### ADTC cohort

Clinical patients from the Mayo Clinic ADTC were used as a third validation cohort (*n* = 264)^34^. All participants underwent a comprehensive clinical evaluation, including a clinical interview, neurological examination, amyloid-PET, MRI, FDG-PET, neuropsychological testing, and *APOE* genotyping, as part of the ADTC diagnostic workup. Amyloid status was based on visual reads by nuclear medicine radiologists with expertise in dementia imaging. Only patients with available pre-treatment FDG-PET were included, and none had received amyloid-reducing therapies at the time of data collection. Clinical diagnoses were established via consensus among neurologists, neuropsychologists, and radiologists with expertise in aging and dementia.

The cohort was clinically heterogeneous and included patients with early symptomatic AD (aMCI or mild AD-type dementia; “AD A + ,” *n* = 138), early symptomatic AD with a high suspicion of co-pathology (e.g., α-synucleinopathy or TDP-43; “AD A+ co-path”, *n* = 23), atypical AD variants (dAD, lvPPA, PCA, motor AD, or behavioral AD; “Atypical AD A + ”, *n* = 64), and non-AD syndromes (e.g., normal pressure hydrocephalus, Parkinson’s disease dementia, vascular dementia, frontotemporal dementia; “Non-AD”). The Non-AD group was further stratified by amyloid status into “Non-AD A + ” (*n* = 11) and “Non-AD A–” (*n* = 28), where Non-AD A+ patients were considered to have a non-AD etiology as the primary driver of symptoms despite having biological AD^46^. A subset of participants (*n* = 28) also had research FTP-PET acquired within six months of FDG-PET as part of concurrent research protocols.

The study was approved by the Mayo Clinic Institutional Review Board and complied with HIPAA privacy regulations. All participants, or their legally authorized representatives, provided written informed consent for the use of their data in research.

### MRI acquisition and processing

MRI scans from the Mayo research cohort and a subset of the ADTC cohort with research MRI (*n* = 28) were acquired on a 3 T GE scanner using a magnetization-prepared rapid gradient echo (MPRAGE) sequence. Acquisition parameters included: repetition time (TR) = 2300 ms, echo time (TE) = 3 ms, flip angle = 8°, field of view (FOV) = 26 cm, reconstructed in a 256 × 256 mm in-plane matrix with a phase FOV of 0.94, and slice thickness = 1.2 mm. MRI scans from the ADNI cohort were acquired on 3 T scanners at individual ADNI sites using standardized MPRAGE protocols (see: https://adni.loni.usc.edu/data-samples/adni-data/neuroimaging/mri/). Parameters included: 1 mm isotropic resolution, TR = 2300 ms, minimum full TE, inversion time = 900 ms, and FOV reconstructed in a 208 × 240 × 256 mm matrix. All MRI images were segmented using the Unified Segmentation algorithm in SPM12, incorporating Mayo Clinic Adult Lifespan Template (MCALT) population-optimized priors and settings. MCALT atlases were subsequently normalized to each individual scan using ANTs.

### Amyloid- and FTP-PET imaging acquisition and processing

Amyloid-PET imaging was performed using [¹¹C]PiB for the Mayo research cohort and [¹⁸F]AV45 (florbetapir) or [^18^F]FBB (florbetaben) for the ADNI cohort. Clinical amyloid-PET imaging in the ADTC cohort was performed using [¹⁸F]AV45 (florbetapir). FTP-PET imaging across all cohorts employed the [¹⁸F]AV1451 (FTP) tracer. Detailed acquisition and processing protocols have been previously described^71,72^ and are available online for the ADNI cohort (https://adni.loni.usc.edu/data-samples/adni-data/neuroimaging/pet/). Research PET images were co-registered to each participant’s corresponding MRI and spatially normalized to the Mayo Clinic Adult Lifespan Template (MCALT) using SPM12. SUVR images were generated by normalizing tracer uptake to the cerebellar crus, and mean SUVR values were extracted from validated meta-regions of interest (meta-ROIs) based on the MCSA202 atlas^72^. Amyloid positivity thresholds were set at >25 centiloid across amyloid ligands^73–76^, except for the ADTC clinical amyloid-PET scans, for which positivity was determined by expert visual reads as described above (and therefore assumed to be >25 centiloid). FTP-PET positivity was defined as SUVR > 1.29^77^

Analyses involving FTP-PET data utilized two parcellations. The Schaefer-400 atlas was applied to train the FDG-PET-based FTP-PET prediction model at the whole-brain level^72^. The CenTauR atlas was used to (1) determine tau stage based on mesial temporal and temporoparietal areas^78,79^ and (2) gather a proxy of tau burden based on the temporal meta-ROI. SUVR values in the CenTauR regions were extracted using the cerebellar reference region and converted to CenTauR values^59^. Tau stages were defined according to the revised criteria for AD diagnosis and staging^35^, as follows: A- (amyloid centiloid ≤25), A (amyloid centiloid ≥25; mesial temporal lobe CenTauR ≤ 39), B (amyloid centiloid ≥25; mesial temporal lobe CenTauR ≥ 39; temporoparietal CenTauR ≤25), C (amyloid centiloid ≥25; temporoparietal CenTauR ≥25 and ≤63), and D (amyloid centiloid ≥25; temporoparietal CenTauR ≥63).

### FDG-PET imaging and processing

FDG-PET imaging in the Mayo research and ADTC cohort was performed using a PET/CT scanner (GE Healthcare) following a 30-minute uptake period during which participants rested in a dimly lit room. Image acquisition lasted 8 min and consisted of four dynamic frames (2 min each), preceded by a low-dose CT transmission scan. In the ADNI cohort, FDG-PET images were acquired as a 30-minute dynamic emission scan composed of six 5-minute frames, following a 30- to 60-minute uptake period. Detailed acquisition protocols for the ADNI cohort are available online (https://adni.loni.usc.edu/data-samples/adni-data/neuroimaging/pet/). FDG-PET images from all cohorts were processed using an MRI-independent pipeline^32^. This included non-linear symmetric diffeomorphic registration to the MCALT, normalization of uptake values to the pons to generate SUVR images, and spatial smoothing with a 6 mm full width at half maximum (FWHM) Gaussian kernel. Regional SUVR values were additionally extracted across the Schaefer-400 atlas for visualization purposes only.

### FDG-PET eigenbrain model

FDG-PET images were analyzed using *StateViewer*, a machine learning–based latent space framework previously developed at Mayo Clinic^32^. This approach applies principal component decomposition to 1000 randomly sampled FDG-PET scans from a large database of clinical and research individuals at Mayo Clinic, and that is non-overlapping with the cohorts of interest in this study. Cohorts of interest (Mayo, ADNI, ADTC) were projected into this latent space, defined by the first 150 components, referred to as “eigenbrains,” to extract participant-level eigenvalues. Each eigenbrain represents an orthogonal axis of spatial metabolic variation across the cortex, ordered by the proportion of variance explained, and each eigenvalue quantifies the degree to which an individual’s metabolic pattern aligns with the spatial topology of that eigenbrain. Each eigenbrain represents an orthogonal axis of spatial metabolic variation across the cortex, ordered by the proportion of variance explained, and each eigenvalue quantifies the degree to which an individual’s metabolic pattern aligns with the spatial topology of that eigenbrain. As such, each participant-level eigenvalue for each eigenbrain represents a mapping onto a specific FDG-PET gradient, resulting in a 1088 ×150 (participant X eigenvalue) table as input.

### Model training in the Mayo research cohort

The FDG-PET-based model of tau prediction is configured as a population-level regression framework. Population-derived regression coefficients are applied to individual participant FDG-PET features to estimate their corresponding regional FTP-PET burden across a standardized brain atlas. We first trained a mixed-effects linear regression framework in the Mayo research cohort to predict regional FTP-PET SUVR across the Schaefer-400 atlas using participant-level FDG-PET eigenvalues. All variables were log-transformed. Model performance was quantified using the coefficient of determination (*R*^2^), adjusted *R*^2^ (*R*^*2adj*^), predicted *R*^2^ (*R*^*2pred*^) obtained via leave-one-out cross-validation across participants, MAE, and MSE. Regressions were iteratively performed with increasing numbers of eigenbrains, and the optimal model was defined as that achieving the highest atlas-wide *R*^*2pred*^. Visual inspection of model performance included brain renderings of *R*^2^ values across participants in each group, and Bland-Altman plots across the Schaefer-400 atlas to assess systematic over- and under-estimation of FTP-PET values at the group- and region-level. In addition, we computed spatial *R*^2^ values between observed and predicted FTP-PET across the Schaefer-400 atlas within each participant, and report the median value aggregated across the cohort. Models were re-run including age, sex, and *APOE* status as covariates to assess potential improvement in prediction, and atlas-wide *R*^2^ values were compared across male and female participants.

To evaluate whether non-linear or regularized machine learning architectures could optimize regional FTP-PET prediction relative to our primary linear model, we benchmarked performance against five alternative regression frameworks: Ridge regression (*α* = 0), Lasso regression (*α* = 1), Elastic Net regression (tuning both *α* and *λ*) Random Forest (implemented via the fast conditional inference forest engine *ranger*), and Support Vector Machines (SVM) utilizing a Radial Basis Function (RBF) kernel. Each algorithm was trained to predict regional log-transformed FTP-PET SUVR values across participants using the optimal number of FDG-PET eigenbrain eigenvalues based on the linear model. Model optimization and hyperparameter tuning were executed via a 5-fold cross-validation. The optimal model was additionally benchmarked against a base model using age, sex, *APOE4* carriership, clinical diagnosis, and CDR-SB as predictors of FTP-PET across participants.

Additional regression models were trained to predict CenTauR mesial temporal, temporoparietal, and temporal meta-ROI values, with the number of eigenbrains determined based on the optimal Schaefer-400 model. CenTauR values were then converted back from a log distribution into their original scale using a Duan’s smearing estimator. They were then submitted to a confusion matrix to derive balanced accuracy, sensitivity, specificity, positive predictive value, and negative predictive value between observed and predicted tau stages. Due to class imbalance, we combined stages A/B (absence of significant tau/mesial temporal tau) and C/B (moderate-to-severe temporoparietal tau). These analyses were performed with and without A- individuals, since this stage is influenced by amyloid status only and not tau.

Additional analyses were performed for clinically relevant case examples, where observed versus predicted tau stages were compared in aAD, aMCI, bvFTD, and CBS, since these syndromes can be either influenced by co-pathologies (e.g., TDP-43 in AD)^36–38^ or result from either AD or other primary proteinopathies (e.g., TDP-43 or tauopathies)^39–42^. Predicted CenTauR values (mesial temporal, temporoparietal, temporal) were also compared between observed stages (A-, A/B, C/D).

### Model validation in the longitudinal Mayo research cohort

Baseline-trained models were applied across participants from the Mayo research cohort with longitudinal FDG-PET and FTP-PET data to compare observed and predicted follow-up Schaefer-400 and CenTauR values. Linear mixed-effects models accounted for within-participant variability and time from baseline. In addition, we computed spatial *R*^2^ values between observed and predicted FTP-PET across the Schaefer-400 atlas for each participant and report the cohort-level median value aggregated across visits. Model performance was summarized using marginal *R*^2^, reflecting variance explained by the fixed effects (predicted tau, time from baseline, and their interaction), marginal MSE, and marginal MAE.

Baseline-trained models on CenTauR values (mesial temporal, temporoparietal, temporal meta-ROI) were applied to extract performance metrics. CenTauR cut-offs were used to determine observed and predicted tau stages for each participant at each time point. First, a confusion matrix was computed for baseline data. Then, another confusion matrix was computed for tau stage transitions (e.g., A/B to C/D) between the first and last visits for each participant. Performance was assessed using balanced accuracy, sensitivity, specificity, positive predictive value, and negative predictive value. Here again, analyses were performed with and without A- individuals.

Sensitivity analyses were performed where baseline-trained models of FTP-PET prediction were re-run after excluding participants who had longitudinal data available. These models were subsequently used to replicate the longitudinal validation analyses listed above. This was done to assess whether any overlap between the training and validation cohorts influenced predictive performance in the longitudinal analyses.

Finally, we conducted a longitudinal validation analysis to determine whether baseline model prediction errors carry prognostic value for future clinical deterioration and disease progression. For each participant, prediction residuals (observed FTP-PET minus predicted FTP-PET) were calculated for the temporal meta-ROI at the baseline visit. Longitudinal clinical progression and downstream tau accumulation were quantified by calculating the change (Δ) in CDR-SB and the change (Δ) in temporal meta-ROI FTP-PET SUVR between the baseline visit and the final follow-up visit. Because clinical scales and residuals often yield non-normal distributions and tied ranks, the association between baseline prediction residuals and subsequent longitudinal changes (Δ CDR-SB and Δ FTP-PET) was evaluated using partial Spearman rank correlation coefficients.

### Model validation in the ADNI cohort

Mayo-derived models were applied to ADNI participants using their *StateViewer* eigenvalues. Linear regression assessed correspondence across the Schaefer-400 and CenTauR values, summarized using median *R*^2^, MAE, and MSE. We computed spatial *R*^2^ values between observed and predicted FTP-PET across the Schaefer-400 atlas for each participant and report the cohort-level median value. A confusion matrix was used to assess agreement between observed and predicted tau stages as described above. Predicted CenTauR temporal meta-ROI values were compared across clinically- and biomarker-defined groups (CU, MCI, Dementia; A + /A-, T + /T-) using ANOVA with Tukey post hoc analyses.

### Model validation in the ADTC cohort

Mayo-derived models were applied to ADTC participants using their *StateViewer* eigenvalues. Linear regression assessed correspondence across the Schaefer-400 and CenTauR values in the subset with available PET-PET, summarized using median *R*^2^, MAE, and MSE. We computed spatial *R*^2^ values between observed and predicted FTP-PET across the Schaefer-400 atlas for each participant and report the cohort-level median value. Agreement between observed and predicted tau stage was not assessed in the ADTC cohort due to the small number of participants with available FTP-PET and significant class imbalance.

Predicted CenTauR values (mesial temporal, temporoparietal, temporal meta-ROI) were compared across clinical groups (AD A + , AD A+ co-path, Atypical AD A + , Non-AD A–, Non-AD A + ) using ANOVA with Tukey post hoc analyses, regardless of FTP-PET availability.

### Reporting summary

Further information on research design is available in the Nature Portfolio Reporting Summary linked to this article.

## Supplementary information

Supplementary Information

Transparent Peer Review file

Reporting Summary

Supplementary information

## Source data

Source Data

## Data Availability

Mayo Clinic data are available under restricted access to ensure patient data confidentiality under HIPAA rules. Requests for data will be granted after review by relevant study team investigators, subject to stipulations of data sharing/data use agreements between Mayo Clinic and other collaborating institutions, and Mayo Clinic and the entity requesting the data. Requests can be submitted using the following link: https://ras-rdrs.mayo.edu and ADNI data can be downloaded at https://adni.loni.usc.edu. The MCALT can be downloaded via https://www.nitrc.org/projects/mcalt/. Source data are provided as a Source Data file. Source data are provided with this paper.

## Code availability

Code used for *StateViewer* and extraction of eigenvalues is referenced in previous work^32^ and is available under the Apache ^1^2.0 license from GitHub. The R codes (version 4.5.2) for statistical analyses can be found using the following Zenodo link: https://doi.org/10.5281/zenodo.22016154.
