# Supplementary material for: A model of brain function to predict tau across Alzheimer’s disease and related disorders
Source: Nat Commun. 2026 Oct 2;17:11302. doi: 10.1038/s41467-026-78300-7 (PMC13724704; doi:10.1038/s41467-026-78300-7)
Supplement: Supplementary file 1 [file 41467_2026_78300_MOESM1_ESM.pdf]

Supplementary Information

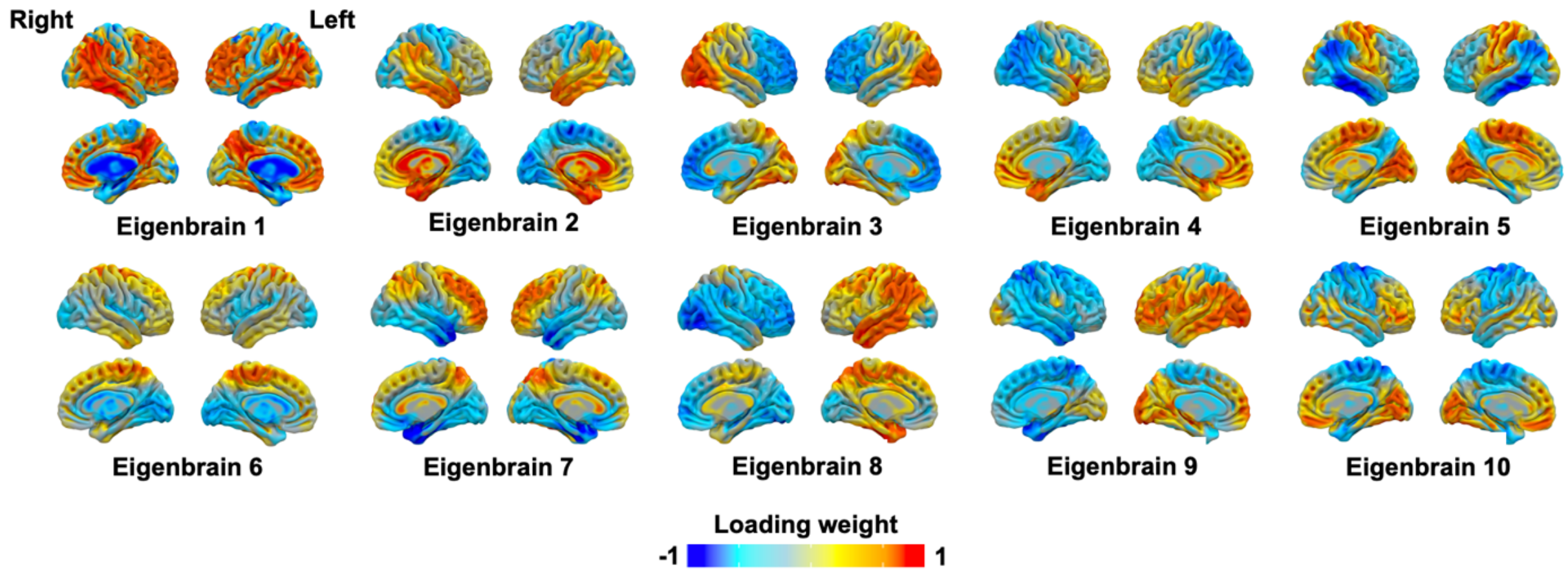

Supplementary Fig. 1. *StateViewer* FDG-PET eigenbrains. The first 10 components (eigenbrains) generated from the principal component decomposition of 1,000 independent FDG-PET images from Mayo Clinic used to develop a machine learning framework called *StateViewer*. The patterns represent relative poles of hypermetabolism (warm colors) and relative hypometabolism (cool colors). Brain renderings have been generated using SurfIce. FDG-PET = Fluorodeoxyglucose positron emission tomography; fMRI = Functional magnetic resonance imaging.

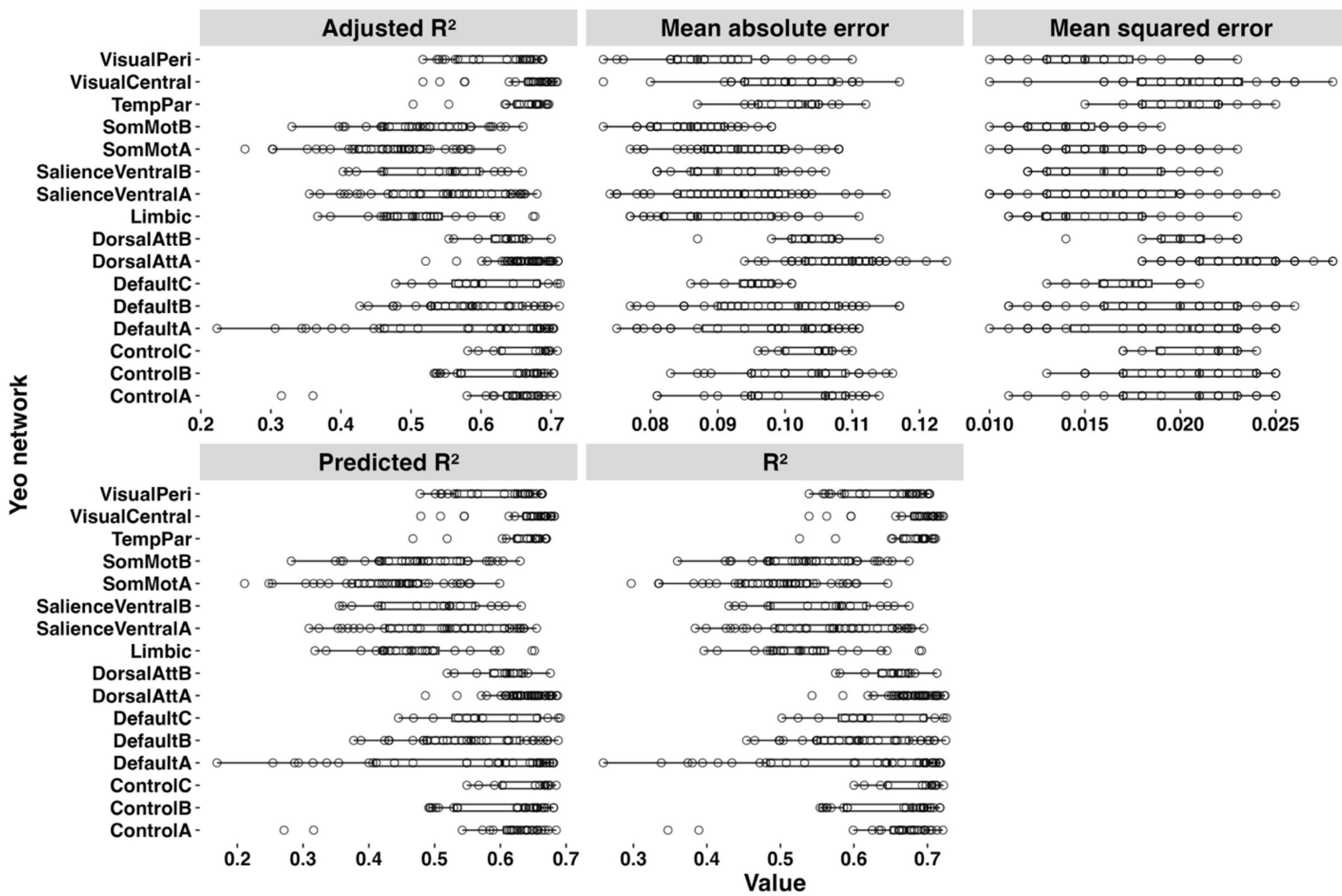

Supplementary Fig. 2. Region- and network-level performance metrics for the Mayo research cohort (training). Performance metrics include *R2*, *R2* adjusted (R^2_{adj}), leave-one-out *R2* predicted (R^2_{pred}), mean absolute error, and mean squared error across all Schaefer-400 regions (represented by dots) and Yeo's 17 networks (represented by rows). Box plots show the median (center line), upper and lower quartiles (box bounds, 25th and 75th percentiles), and minimum to maximum values within 1.5× the interquartile range (whiskers). Source data are provided as a Source Data file.

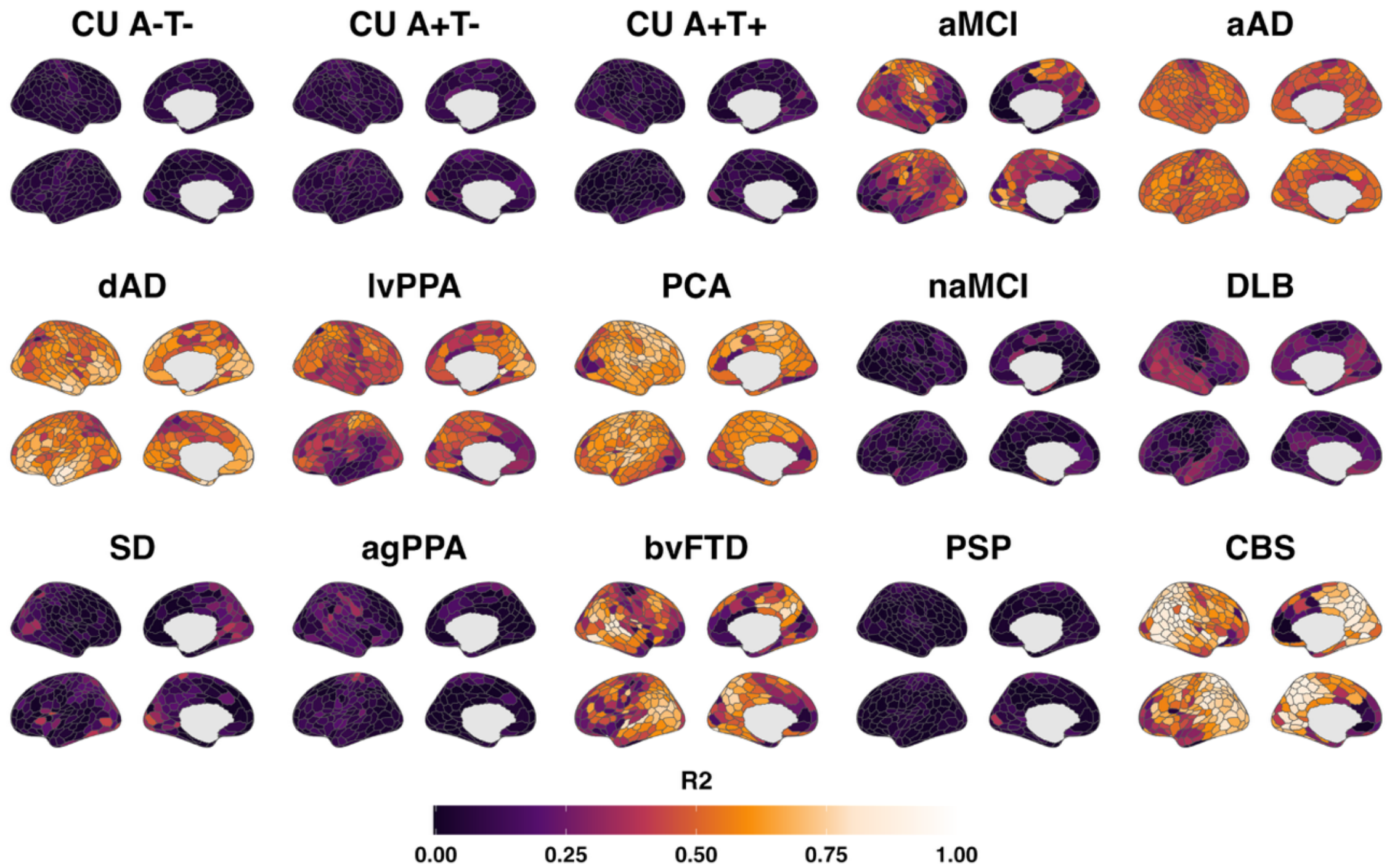

Supplementary Fig. 3. Group-wise distributions of R^2 values across the Schaefer-400 atlas in the Mayo research cohort. Brain renderings were generated using the *R* package ggseg. Source data are provided as a Source Data file. A+ = Amyloid-positive; A- = Amyloid-negative; aAD = Amnestic Alzheimer's disease; agPPA = Agrammatic primary progressive aphasia; aMCI = Amnestic mild cognitive impairment; bvFTD = Behavioral variant frontotemporal dementia; CBS = Corticobasal syndrome; CU = Cognitively unimpaired; dAD = Dysexecutive Alzheimer's disease; DLB = Dementia with Lewy Bodies; FDG-PET = Fluorodeoxyglucose positron emission tomography; lvPPA= Logopenic variant primary progressive aphasia; naMCI = Non-amnestic MCI; PSP = Progressive supranuclear palsy; SD = Semantic dementia; T+ = Tau-positive; T- = Tau-negative.

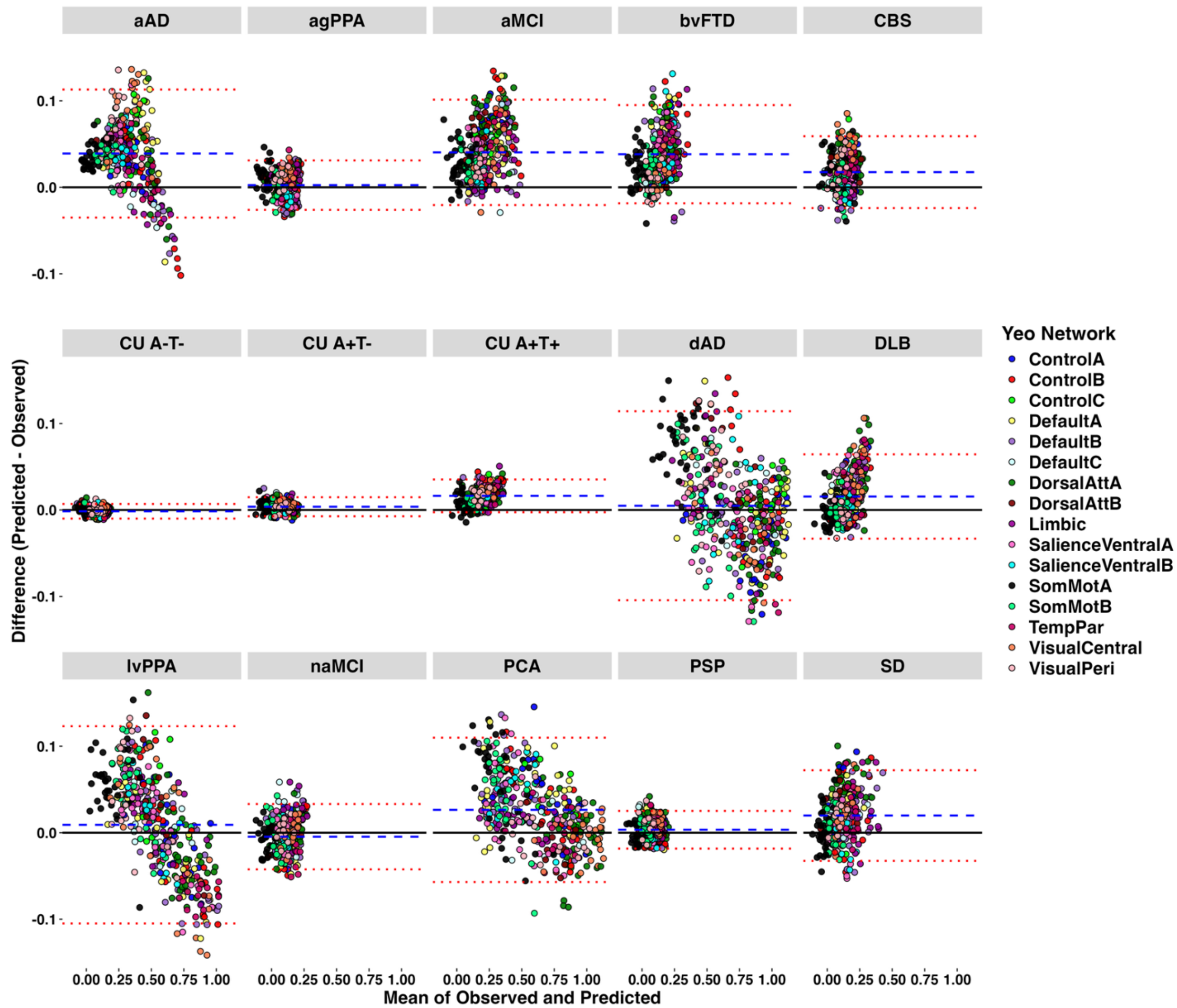

Supplementary Fig. 4. Bland-Altman plots for region-wise predictions across the Schaefer-400 atlas in the Mayo research cohort. Each dot is a region of the Schaefer-400 atlas, color-coded by Yeo's networks. The blue dash lined represents the mean difference between observed and predicted values, surrounded by 95% intervals represented by the red dotted lines. A blue line below the solid black line indicates systematic underestimation, and a blue line above the solid black line indicates systematic overestimation. Source data are provided as a Source Data file. A+ = Amyloid-positive; A- = Amyloid-negative; aAD = Amnestic Alzheimer's disease; agPPA = Agrammatic primary progressive aphasia; aMCI = Amnestic mild cognitive impairment; bvFTD = Behavioral variant frontotemporal dementia; CBS = Corticobasal syndrome; CU = Cognitively unimpaired; dAD = Dysexecutive Alzheimer's disease; DLB = Dementia with Lewy Bodies; FDG-PET = Fluorodeoxyglucose positron emission tomography; FTP= Flortaucipir; lvPPA= Logopenic variant primary progressive aphasia; naMCI = Non-amnestic MCI; PSP = Progressive supranuclear palsy; SD = Semantic dementia; T+ = Tau-positive; T- = Tau-negative.

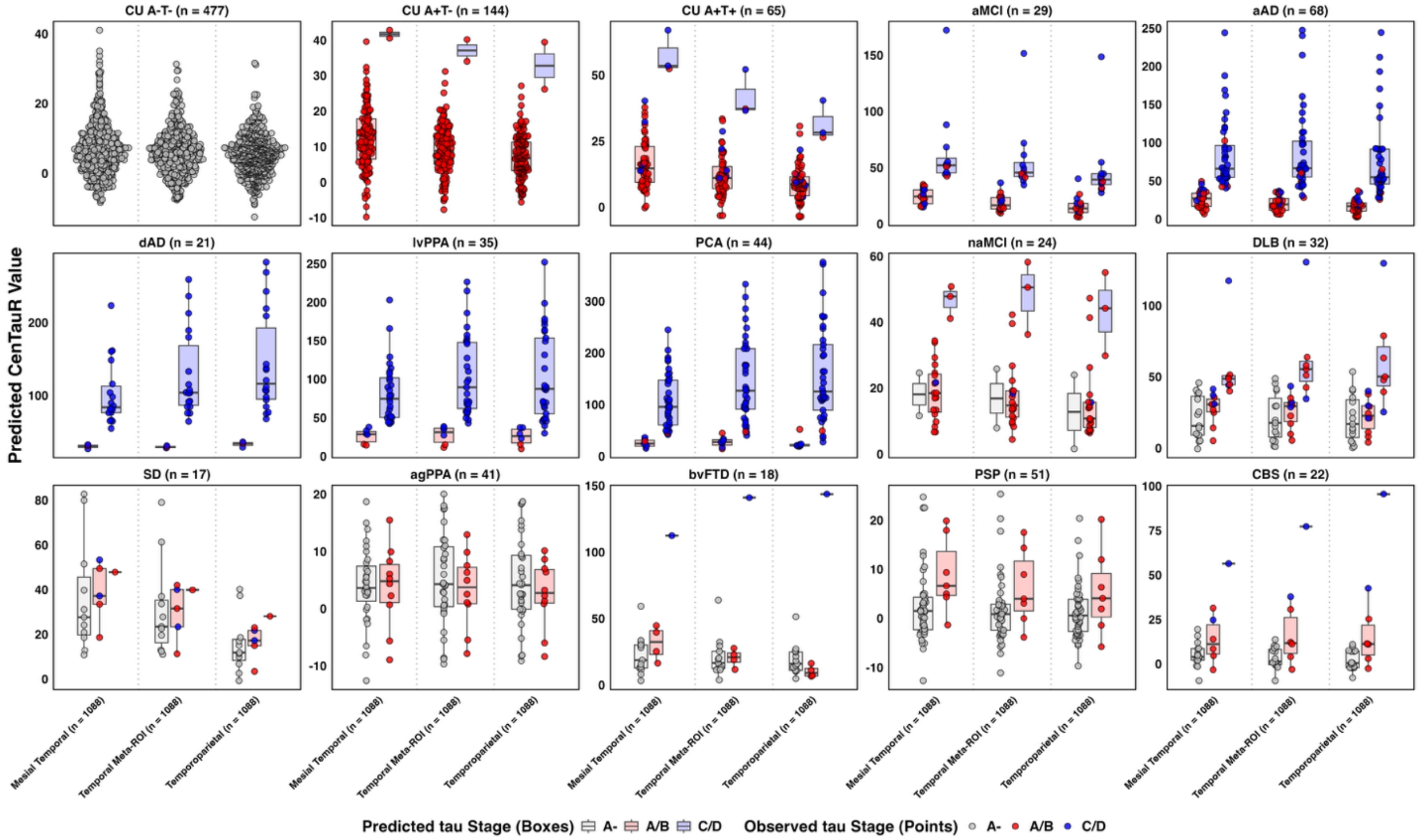

Supplementary Fig. 5. Group comparisons in CenTauR values in the Mayo research cohort stratified by tau stage. Each dot is a participant, color-coded by observed tau stage (dots) and predicted tau stage (box). Box plots show the median (center line), upper and lower quartiles (box bounds, 25th and 75th percentiles), and minimum to maximum values within 1.5× the interquartile range (whiskers). No statistical analysis has been performed, as these data are presented for visualization purposes only. Source data are provided as a Source Data file. A+ = Amyloid-positive; A- = Amyloid-negative; aAD = Amnestic Alzheimer's disease; agPPA = Agrammatic primary progressive aphasia; aMCI = Amnestic mild cognitive impairment; bvFTD = Behavioral variant frontotemporal dementia; CBS = Corticobasal syndrome; CU = Cognitively unimpaired; dAD = Dysexecutive Alzheimer's disease; DLB = Dementia with Lewy Bodies; lvPPA= Logopenic variant primary progressive aphasia; naMCI = Non-amnestic MCI; PSP = Progressive supranuclear palsy; SD = Semantic dementia; T+ = Tau-positive; T- = Tau-negative.

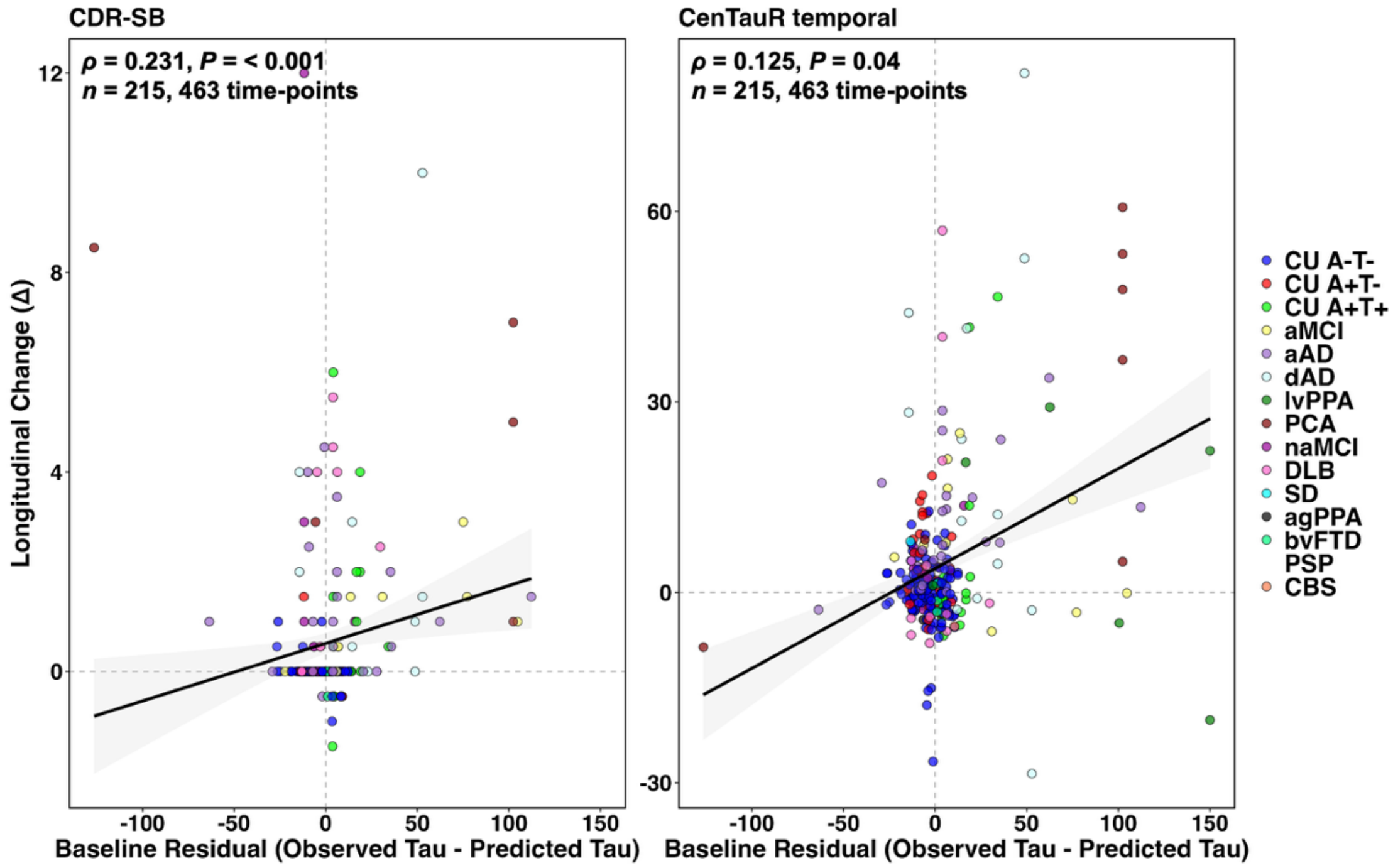

Supplementary Fig. 6. Relationships between baseline FTP-PET residuals and change in CDR-SB and CenTauR temporal meta-ROI in the Mayo longitudinal cohort. Each dot is a participant, color-coded by diagnosis. A value higher than the vertical 0 line indicates a lower predicted FTP-PET value than predicted, and vice-versa. A higher value on the Y-axis indicates a greater change in CDR-SB or temporal meta-ROI, and vice-versa. The grey area around the fitted line indicated 95% confidence intervals. Source data are provided as a Source Data file. A+ = Amyloid-positive; A- = Amyloid-negative; aAD = Amnestic Alzheimer's disease; agPPA = Agrammatic primary progressive aphasia; aMCI = Amnestic mild cognitive impairment; bvFTD = Behavioral variant frontotemporal dementia; CBS = Corticobasal syndrome; CU = Cognitively unimpaired; dAD = Dysexecutive Alzheimer's disease; DLB = Dementia with Lewy Bodies; FDG-PET = Fluorodeoxyglucose positron emission tomography; FTP= Flortaucipir; lvPPA= Logopenic variant primary progressive aphasia; ROI = Region of interest; naMCI = Non-amnestic MCI; PSP = Progressive supranuclear palsy; SD = Semantic dementia; T+ = Tau-positive; T- = Tau-negative.

Supplementary Table 1 Comparison of alternative models in the Mayo research cohort

Model	R^2	MSE	MAE
Linear	0.71 [0.61-0.77]	0.01 [0.01-0.02]	0.09 [0.08-0.09]
Linear with age, sex, and *APOE*	0.71 [0.62-0.78]	0.01 [0.01-0.02]	0.09 [0.08-0.09]
Elastic Net	0.65 [0.54-0.73]	0.13 [0.12-0.14]	0.09 [0.09-0.10]
Lasso	0.65 [0.54-0.73]	0.13 [0.12-0.14]	0.09 [0.09-0.10]
Random Forest	0.59 [0.49-0.59]	0.15 [0.13-0.16]	0.10 [0.09-0.11]
Ridge	0.65 [0.54-0.73]	0.13 [0.12-0.14]	0.09 [0.09-0.10]
SVM	0.64 [0.52-0.72]	0.13 [0.12-0.14]	0.09 [0.09-0.10]
Linear base model*	0.51 [0.40-0.60]	0.13 [0.12-0.14]	0.09 [0.08-0.09]

*Using age, sex, *APOE4* carriership, clinical diagnosis clinical dementia rating scale sum-of-boxes as predictors

Supplementary Table 2 Confusion matrix results for the Mayo research cohort

Confusion matrix without A- individuals	
Metric	Value
Sensitivity	0.94
Specificity	0.79
Positive predictive value	0.89
Negative predictive value	0.87
Prevalence	0.65
Detection rate	0.60
Detection prevalence	0.68
Balanced accuracy	0.86

Confusion matrix with A- individuals			
Metric	A-	A/B	C/D
Sensitivity	1.00	0.94	0.79
Specificity	1.00	0.95	0.98
Positive predictive value	1.00	0.89	0.87
Negative predictive value	1.00	0.87	0.96
Prevalence	0.56	0.29	0.16
Detection rate	0.56	0.27	0.12
Detection prevalence	0.56	0.30	0.14
Balanced accuracy	1.00	0.95	0.88

A- = Amyloid-negative.

Supplementary Table 3 Confusion matrix results for case examples in the Mayo research cohort (aMCI, aAD, bvFTD, CBS)

Confusion matrix without A- individuals

Metric	Value
Sensitivity	0.86
Specificity	0.78
Positive predictive value	0.76
Negative predictive value	0.87
Prevalence	0.45
Detection rate	0.39
Detection prevalence	0.51
Balanced accuracy	0.82

Confusion matrix with A- individuals

Metric	A-	A/B	C/D
Sensitivity	1.00	0.76	0.87
Specificity	1.00	0.92	0.84
Positive predictive value	1.00	0.86	0.78
Negative predictive value	1.00	0.85	0.91
Prevalence	0.20	0.40	0.39
Detection rate	0.20	0.31	0.34
Detection prevalence	0.20	0.36	0.44
Balanced accuracy	1.00	0.84	0.86

A- = Amyloid-negative; aMCI = amnestic mild cognitive impairment; aAD = amnestic Alzheimer's disease; bvFTD = behavioral variant frontotemporal dementia; CBS = corticobasal syndrome.

Supplementary Table 4 Confusion matrix results for the Mayo longitudinal cohort

Baseline confusion matrix without A- individuals

Metric	Value
Sensitivity	0.96
Specificity	0.64
Positive predictive value	0.87
Negative predictive value	0.86
Prevalence	0.71
Detection rate	0.67
Detection prevalence	0.78
Balanced accuracy	0.80

Baseline confusion matrix with A- individuals

Metric	A-	A/B	C/D
Sensitivity	1.00	0.87	0.86
Specificity	1.00	0.98	0.95
Positive predictive value	1.00	0.96	0.64
Negative predictive value	1.00	0.93	0.98
Prevalence	0.56	0.34	0.10
Detection rate	0.56	0.30	0.08
Detection prevalence	0.56	0.31	0.13
Balanced accuracy	1.00	0.92	0.90

Longitudinal confusion matrix without A- individuals

Metric	A/B to A/B	A/B to C/D	C/D to C/D
Sensitivity	0.90	0.17	0.86
Specificity	0.85	0.94	0.88
Positive predictive value	0.92	0.29	0.67
Negative predictive value	0.82	0.89	0.96
Prevalence	0.65	0.13	0.22
Detection rate	0.59	0.02	0.19
Detection prevalence	0.64	0.07	0.29
Balanced accuracy	0.88	0.55	0.87

Longitudinal confusion matrix with A- individuals

Metric	A- to A-	A/B to A/B	A/B to C/D	C/D to C/D
Sensitivity	1.00	0.89	0.17	0.86
Specificity	1.00	0.97	0.97	0.95
Positive predictive value	1.00	0.91	0.29	0.67
Negative predictive value	1.00	0.96	0.95	0.98
Prevalence	0.56	0.27	0.06	0.10
Detection rate	0.56	0.24	0.01	0.09
Detection prevalence	0.56	0.26	0.03	0.13
Balanced accuracy	1.00	0.93	0.57	0.90

A- = Amyloid-negative.

Supplementary Table 5 Confusion matrix results for the ADNI cohort

Confusion matrix without A- individuals	
Metric	Value
Sensitivity	0.87
Specificity	0.65
Positive predictive value	0.79
Negative predictive value	0.77
Prevalence	0.60
Detection rate	0.52
Detection prevalence	0.66
Balanced accuracy	0.76

Confusion matrix with A- individuals			
Metric	A-	A/B	C/D
Sensitivity	1.00	0.79	0.77
Specificity	1.00	0.94	0.91
Positive predictive value	1.00	0.87	0.65
Negative predictive value	1.00	0.89	0.95
Prevalence	0.47	0.35	0.18
Detection rate	0.47	0.28	0.14
Detection prevalence	0.47	0.32	0.21
Balanced accuracy	1.00	0.86	0.84

A- = Amyloid-negative.
