# Supplementary material for: A model of brain function to predict tau across Alzheimer’s disease and related disorders
Source: Nat Commun. 2026 Oct 2;17:11302. doi: 10.1038/s41467-026-78300-7 (PMC13724704; doi:10.1038/s41467-026-78300-7)
Supplement: Supplementary file 2 [file 41467_2026_78300_MOESM2_ESM.pdf]

nature portfolio

Peer Review File

A model of brain function to predict tau across Alzheimer's disease and related disorders

Corresponding Author: Dr David Jones

This file contains all reviewer reports in order by version, followed by all author rebuttals in order by version.

Version 0:

Reviewer comments:

Reviewer #1

(Remarks to the Author)
This work by Corriveau-Lecavalier et al. applies eigenbrain approaches to predict tau PET from FDG PET. The prediction performances are impressive, supported by rigorous validations across longitudinal, external, and clinically heterogeneous samples. The manuscript is well-written, and the overarching research question, understanding in vivo tau pathology patterns, is a central topic in ADRD research. The manuscript would benefit substantially from demonstrating the clinical or scientific value of the proposed approach beyond prediction accuracy metrics, which I believe would markedly strengthen its impact. My comments are as follows:

Main Comments:

1. Validations of clinical impacts of this approach would be appreciated. While the prediction performances reported in this manuscript are impressive, the clinical rationale for the proposed approach is not clearly established, either in the motivation or through experiments. In current memory clinic practice, how frequently do patients presenting with dementia or cognitive impairment undergo FDG PET, and under what conditions would FDG PET be performed instead of—or prior to—tau PET? Clarifying this clinical context would strengthen the argument for the approach's practical utility. Furthermore, for non-AD primary tauopathies (e.g., PSP, CBS, or FTLD-tau) or clinically heterogeneous populations, it would be worth examining whether FDG PET-predicted tau values, when integrated with established biomarkers (e.g, p-tau biomarkers), improve differential diagnosis compared to either modality alone.

2. Post-hoc experiments for exploring the predicted patterns would greatly enhance the impact. The authors claim that "the multimodal prediction of tau-PET patterns across the ADRD spectrum is critical for advancing our understanding of tau pathology and its relationship to large-scale functional dynamics and neurodegeneration." However, the analyses presented do not substantively support this claim. For instance, do systematic overestimation or underestimation of tau burden from FDG PET reflect meaningful metabolic signals, such as hypometabolism disproportionate to tau load, or vice versa. If found, that could yield novel insights into the metabolism–tau relationship. Given that the authors have access to longitudinal FDG and tau PET data, exploring how prediction residuals or temporal changes in prediction accuracy relate to disease progression or clinical trajectories could provide novel mechanistic insights that go beyond the prediction framework itself. For example, fast tau-accumulation or low tau-accumulation and its corresponding predictions from FDG-PET at baseline?

3. Distinguishment between translation and simulation. The authors note that network-based approaches achieved only modest performance in predicting tau burden. However, it is important to clarify that most such models are designed to simulate tau propagation dynamics from putative epicenters, where individual-level input data serve primarily as constraints on model behavior. These approaches are typically not intended for individual-level prediction. In contrast, the present work performs a cross-modal translation from FDG PET to tau PET, leveraging spatially rich individual-level metabolic information alongside shared vulnerability properties across brain networks. Given these fundamental differences in objective and input structure, the superior prediction performance observed here is expected, and the comparison with network-based simulation approaches should be framed accordingly to avoid a misleading implied equivalence.

4. Non-linear prediction approaches. The authors employ linear models for prediction, which yield impressive results and have the advantage of straightforward interpretability. However, the relationship between regional glucose metabolism and tau burden is non-linear, particularly across disease stages and diagnostic categories. It would be informative to compare

the current linear approach against non-linear alternatives—such as Random Forest regression, which retains high interpretability via feature importance—or recently developed tabular foundation models such as TabPFN (Hollmann et al., 2025, Nature). If non-linear models yield substantially improved performance, this would be an important finding in itself; if they do not, it would provide useful evidence supporting the linearity assumption and further validate the current approach.

Minor Comments:
1. The Mayo research cohort comprises three sub-cohorts acquired across multiple sites. Please clarify whether harmonization procedures (e.g., ComBat or equivalent methods) were applied to account for potential site-related confounds in PET measurement.

2.In the scatter plots of Figures 4, 6, and 7, adding an identity line (y = x) would facilitate visual assessment of prediction bias. Specifically, systematic over or underestimation of tau values, relative to observed measurements.

3. It would be informative to examine whether prediction performance differs by sex, given the influence of biological sex on AD pathology.

Reviewer #2

(Remarks to the Author)
This paper presents a machine learning framework for predicting tau-PET burden across the Alzheimer's disease and related disorders (ADRD) spectrum using FDG-PET imaging. The authors leverage StateViewer — a pre-existing clinical decision support system built on principal component decomposition of 1,000 FDG-PET scans — to extract participant-level eigenvalues ("eigenbrains") that serve as predictors in a linear regression model targeting regional tau-PET uptake across the Schaefer-400 atlas. The model was trained on a large Mayo Clinic research cohort (n = 1,069) spanning normal aging, Alzheimer's disease (AD), dementia with Lewy bodies (DLB), and frontotemporal dementia (FTD); a major strength of the study. The optimal model incorporated 50 eigenbrains. Three independent validation experiments on diverse data were conducted, for which the author should be commended. The model demonstrated high training-set performance (median R^2pred = 0.58 at participant level; R^2 = 0.93 at region level) and achieved 84–95% accuracy in tau staging across cohorts.

Strengths:
1. Large training dataset spanning across AD and other neurodegenerative diseases, as well as multiple independent validation sets.
2. FDG-PET is closely related to tau pathology and has great clinical utility. Predicting tau burden and progression using FDG-PET complements the network/epicenter-based framework.
3. The tau staging confusion matrices and the case example in amnestic AD phenotypes (aMCI/aAD) ground the model's value in practical diagnostic scenarios.

Major comments:
While the authors addressed several important limitations in the manuscript, some of which need to be evaluated.
1. The individual-level prediction is misleading given the model's design and the evaluation metrics. The model is fundamentally a population-level regression framework, not an individual brain model. For each of the 400 Schaefer regions, a separate linear regression is trained across all 1,069 participants, using their FDG-PET eigenvalues as predictors. When applied to a new individual, population-derived regression coefficients are used to predict where that person falls on the group regression line. Two individuals with identical FDG-PET eigenvalue profiles would receive identical tau predictions, regardless of individual variation in their FDG-PET–tau relationship due to co-pathologies, genetic factors, or metabolic individuality (ie, it is not individual brain modeling in a mechanistic sense).
2. The paper repeatedly describes its R^2 values as "participant-level" metrics, but this label is misleading. What is actually being computed is, for each brain region, the proportion of variance in tau across participants explained by the model, with the median taken across the 400 regions (IQR reflecting variability across regions). The R^2pred = 0.58, therefore, tells the reader: across 400 regions, the median proportion of variance in tau levels across participants explained by the model is 58%. This is a population-level cross-sectional correlation, not an index of how accurately the model predicts any given individual's tau distribution. True individual-level evaluation would require computing, within each participant, the spatial correlation between their 400-element observed tau vector and their 400-element predicted tau vector — an individual-level spatial R^2 across brain regions. The authors should show the distributions of individual-level correlations and MSEs, or at the very least adjust the tone of individual predictions across the results and interpretation/discussion.
3. Another substantive methodological problem in the paper is the overlap between the longitudinal validation cohort and the training set. A re-analysis using only participants whose baseline data were withheld from training would provide a cleaner estimate of temporal generalizability. At least, a sensitivity analysis that excludes longitudinal participants from the training set would be helpful.
4. As mentioned in the study, the tau-PET tracer used does not typically bind to non-AD tauopathies. It's not clear what the meaning of applying tau stages to non-AD cases is.
5. FDG-PET hypometabolism is likely largely a downstream consequence of tau-related neurodegeneration in AD. The model is framed as predicting tau from FDG-PET, but the causal relationship likely runs in both directions, or from tau to hypometabolism. The claim that FDG-PET gradients constitute a "blueprint" for tau accumulation, rather than a mirror or downstream effect of it, is a strong causal inference that the cross-sectional correlational design cannot support.
6. Figs 3 and 6. It would be more interesting to show the real/observed tau-PET maps (or differences) alongside the predicted maps across the cohorts.
7. Fig 5A. It is not clear what the subject-level error in prediction (qualitatively and more importantly quantitatively).

Minor:
8. The abstract results are a bit vague in some instances without quantification or details, eg: “The model accurately predicted both the magnitude and spatial distribution of taupathology at the individual and regional levels across cohorts” Or “generation of biologically plausible tau patterns in a clinical cohort without tau-PET”
9. Figure 2B shows a greater hypometabolism towards more red color. But more hypometabolism is associated with lower FDG-PET SUVR values. Either inverse the color or change the colorbar scale label to hypometabolism rather than FDG-PET SUVR.
10. Fig 4A, ideally results for more ROIs should be shown and their corresponding correlations/R^2. B) What is the recall/precision of the model for this staging?
11. No stats are included in the majority of the figures/figure captions
12. There are multiple brains overlapping in the Eigenbrain 7 in Supp Fig1.

Reviewer #3

(Remarks to the Author)
The manuscript by Corriveau-Lecavalier et al describes an FDG-PET and brain function informed machine learning model to predict regional tau-PET SUVRs across a range of older adults, individuals with AD at different clinical levels of severity, and individuals with non-AD neurodegenerative diseases. The introduction is well-written, and many of the relevant papers are cited. The description of brain networks, gradients, and their relationship to function and cognition are appropriate. As someone who was not familiar with StateViewer before reading this manuscript, I nonetheless found the methods (and Figure 1) sufficiently clear. The manuscript uses independent samples for replication and a “longitudinal” replication as well, and they correctly acknowledge the limitations of this latter sample. I think the statistical analyses are largely appropriate, however, I was left with many questions about model performance and characteristics that were unanswered (see below). These made it difficult to evaluate some of the study’s conclusions. Overall, their model shows FDG-PET can predict late-stage tau pathology measured by [18F]flortaucipir, with regional patterns of flortaucipir uptake that correspond well to known uptake patterns in a number of different AD syndromes (aAD, dAD, PCA, lvPPA). I have a few concerns, as well as some minor suggestions further below.

Major:
Missing from this model is replication with a non-flortaucipir tau-PET tracer (I acknowledge that such a dataset with FDG-PET and a non-FTP tau-PET tracer may not exist). Because of flortaucipir’s lower sensitivity to tau and higher levels of noise (Ghatamaneni et al Acta Neuropathologica 2025), one wonders about whether FDG is able to capture the broader dynamic range of tau pathology that can be captured with other tau-PET imaging agents. In this connection, FDG may be better-suited in predicted tau that has reached the detection threshold of Flortaucipir, but perhaps less useful at detecting lower levels, which is especially relevant in preclinical disease. On a related note, some tau-PET agents such as PI2620 have been reported to bind to non-AD tauopathies, especially PSP 4R tau and to a lesser extent CBD 4R tau. How do the authors suggest their model would generalize under these circumstances? Perhaps taking these limitations together, if a dataset without FDG and a different tau-PET ligand does not exist, it would be better to call the maps “predicted Flortaucipir maps” rather than tau-PET, as some elements of the results may not generalize to other tau-PET ligands.

Regarding the study’s main conclusions, can the authors please elaborate on how this study advances the mechanistic insight into neurodegeneration and tau-PET (mentioned in abstract and discussion)? I agree with the authors that some pathologies accumulate preferentially within specific networks/gradients, and that some clinical syndromes are more likely to be associated with specific pathologies. But I don’t see how the present manuscript’s model helps explain anything mechanistic about vulnerability/resilience of any specific network. Because of the large amount of neurodegenerative tauopathies in which there is an FDG-Flortaucipir dissociation (~40% of bvFTD, ~50% of CBS, most PSP, most agPPA cases are 3R or 4R tauopathies), I do not understand how the model provides mechanistic insights into the relationship between tau and neurodegeneration.

Another important missing element, in my opinion, is the ability to predict tau in more clinically/radiographically challenging scenarios: In CBS, for example, where AD accounts for ~50% of CBS cases, how can an FDG-based model predict which cases are caused by AD-type tau? This is a clinically important question that remains unaddressed. CBS is not included in the present study, but CBS data is reported in the Stateviewer manuscript, and I wonder why these results are not reported. Can the authors show side-by-side display of predicted vs observed tau-PET in the training and validation cohorts? As currently displayed, while Figure 3 recapitulates expected relationships between neurodegenerative clinical syndromes and patterns of tau-PET, without seeing the observed data, these brains are not informative. While I appreciate that Supplementary Figure 3 shows this in scatterplot format, I suggest reporting R2 values for each predicted-observed pair. Perhaps even better could be displaying R2 brain-maps. Bland-Altman plots showing the difference between predicted and observed tau-PET, with colours highlighted similar to figure 4 would be helpful to present.
Moreover, can the authors report observed vs predicted tau-PET in the medial temporal (perhaps entorhinal cortex and amygdala) and temporal meta-ROI for the ADNI and Mayo cohorts?

The authors combined stages A&B and stages C&D because of class imbalance problems, which is understandable. I am concerned that this creates an artificially “easy” task of distinguishing between those with little-to-no detectable tau vs those with moderate-to-severe tau. The results mention that the FDG-PET model was not improved by the addition of covariates.

However, I wonder how much better this advanced model is compared to a base model of age, sex, APOE4 genotype, clinical diagnosis and MOCA/CDR-SoB.

The authors elected not to try to predict tau in A- individuals. However, to me, this misses an important opportunity for showing the model predicts “true negatives”. It is important to see how the FDG-PET and brain function model can predict tau-PET stages in non-AD diseases, and the accurate prediction of little-to-no tau-PET (here referring to 3R&4R PHF tau) is a highly relevant clinical scenario (I suspect based the model will be able to do this well). Can the authors please report the results of these analyses?

Can the authors please state whether any other individual-level data was used in the model? In the methods, I think the explanation of how the FDG-PET data are used in the model is well-explained, however, I am less clear about the model uses brain function: does this refer to the relationship of FDG-PET signal mapping onto brain networks/gradients associated with cognition and behaviour?
Figure 4B: Can the authors please present a similar plot for MTL tau? Also, because many readers (myself included) do not yet have a strong intuition regarding CenTauR values, can you please display FTP SUVRs on the right y-axis?

Figure 5: In my view, the analyses on stage progression, are interesting and important; I just have a hard time interpreting them without knowing more basic performance. Can you please display a 4x4 matrix with baseline observed vs predicted tau-PET stages? Can the manuscript report overall accuracy of stage transitions that do not include the A- group? The inclusion of the large A- group can artificially increase accuracy statistics.

Is it possible the R2 statistics are inflated by the large proportion of CU individuals, who have very little to no tau-PET?

Minor:
Please mention in the limitations that ADNI is heavily pre-screened and has a much lower level of co-pathology than other cohorts. It would be interesting to see replication in a more diverse setting.

Supplementary Fig 2: Perhaps I have misunderstood, but I do not see where or how the Yeo 17 network masks are evaluated/displayed.

Supplementary figure 3: Can the line of identity please be shown to facilitate interpretation?

Version 1:

Reviewer comments:

Reviewer #1

(Remarks to the Author)
The authors have satisfactorily addressed all of my previous concerns. The additional experiments substantially strengthen the manuscript and significantly enhance its scientific value. I have no further comments.

Reviewer #2

(Remarks to the Author)
The authors have addressed my comments. Thank you.

Reviewer #3

(Remarks to the Author)
The authors have done an impressive and thorough job at answering my critiques. I have no further comments. Congratulations on this important manuscript.

Open Access This Peer Review File is licensed under a Creative Commons Attribution 4.0 International License, which permits use, sharing, adaptation, distribution and reproduction in any medium or format, as long as you give appropriate credit to the original author(s) and the source, provide a link to the Creative Commons license, and indicate if changes were made.
In cases where reviewers are anonymous, credit should be given to 'Anonymous Referee' and the source.
The images or other third party material in this Peer Review File are included in the article's Creative Commons license, unless indicated otherwise in a credit line to the material. If material is not included in the article's Creative Commons license and your intended use is not permitted by statutory regulation or exceeds the permitted use, you will need to obtain permission directly from the copyright holder.
To view a copy of this license, visit https://creativecommons.org/licenses/by/4.0/

RESPONSE TO REVIEWERS' COMMENTS

Reviewer #1 (Remarks to the Author):

This work by Corriveau-Lecavalier et al. applies eigenbrain approaches to predict tau PET from FDG PET. The prediction performances are impressive, supported by rigorous validations across longitudinal, external, and clinically heterogeneous samples. The manuscript is well-written, and the overarching research question, understanding in vivo tau pathology patterns, is a central topic in ADRD research. The manuscript would benefit substantially from demonstrating the clinical or scientific value of the proposed approach beyond prediction accuracy metrics, which I believe would markedly strengthen its impact. My comments are as follows:

Main Comments:

1. Validations of clinical impacts of this approach would be appreciated. While the prediction performances reported in this manuscript are impressive, the clinical rationale for the proposed approach is not clearly established, either in the motivation or through experiments. In current memory clinic practice, how frequently do patients presenting with dementia or cognitive impairment undergo FDG PET, and under what conditions would FDG PET be performed instead of—or prior to—tau PET? Clarifying this clinical context would strengthen the argument for the approach's practical utility. Furthermore, for non-AD primary tauopathies (e.g., PSP, CBS, or FTLD-tau) or clinically heterogeneous populations, it would be worth examining whether FDG PET-predicted tau values, when integrated with established biomarkers (e.g, p-tau biomarkers), improve differential diagnosis compared to either modality alone.

We thank the reviewer for their positive comments and the opportunity to clarify the clinical rationale and practical utility of our approach. In current clinical practice, especially in North American tertiary and academic memory centers, FDG-PET is a widely available, routinely reimbursed, and established imaging modality for evaluating cognitive impairment. Conversely, tau-PET remains mostly constrained to research settings or specialized clinical trials due to limited reimbursement, high cost, and restricted geographic availability.

FDG-PET has many utilities that are complementary or distinct to tau-PET and that can justify its ordering prior to or instead of tau-PET, including clarifying etiologic and differential diagnosis, index and track disease biology severity, and estimate the presence of co-pathologies. By leveraging FDG-PET to estimate the presence and distribution of AD-type tau, our model provides a highly scalable surrogate that maximizes the clinical and research yield of an existing imaging modality.

We have added language in-text to expand on this topic, as follows:

Introduction (page 3): "*In this study, we developed a scalable and interpretable model of AD-type tau prediction based on FDG-PET. We selected FDG-PET as our primary imaging modality because it is currently utilized in clinical practice for the evaluation of degenerative cognitive disorders to clarify differential diagnosis, index disease severity, and track progression.*"

Discussion (page 16): "*Overall, our model provides a highly scalable surrogate that maximizes the clinical and research yield of FDG-PET, and supports the role of this imaging modality as a close proxy of AD-type tau accumulation, related neurodegeneration, and clinical symptoms.*"

Given this use case, we also completely agree with the reviewer that examining our model within clinically heterogeneous populations and non-AD groups is crucial for establishing its specificity and clinical utility. To directly address this, we conducted additional analyses in clinically relevant groups that can be caused either by AD or other primary etiologies, i.e., bvFTD and CBS (in addition to the analyses on aMCI/aAD). Our model achieved strong classification accuracy (82-85%) for tau staging within these challenging groups, demonstrating that it successfully avoids

predicting a high FTP-PET burden in non-AD disorders despite the presence of FDG-PET hypometabolism. These findings are detailed on page 11 and visualized on Figure 4.

Regarding the integration of plasma biomarkers: while we agree this represents a promising future diagnostic workflow, the primary objective of the current study was to develop a standalone imaging surrogate optimized to predict the presence and spatial extent of AD-type tau. Intentionally keeping the model independent of fluid biomarkers ensures its maximum generalizability, particularly in clinical settings or retrospective cohorts (such as our ADTC cohort) where plasma biomarkers are unavailable.

We have expanded our manuscript to elaborate on these additional analyses and have added text to the Discussion to highlight the future potential of multi-biomarker integration:

Discussion (page 17): "*Finally, an important future direction of this work is the assessment of integration FDG-PET markers and plasma biomarkers into minimal, cost-effective clinical workflow for diagnostic purposes. While our standalone imaging model provides robust utility when fluid biomarkers are missing, combining spatial metabolic predictions with highly accessible plasma assays could further refine differential diagnosis in complex clinical settings.*"

2. Post-hoc experiments for exploring the predicted patterns would greatly enhance the impact. The authors claim that "the multimodal prediction of tau-PET patterns across the ADRD spectrum is critical for advancing our understanding of tau pathology and its relationship to large-scale functional dynamics and neurodegeneration." However, the analyses presented do not substantively support this claim. For instance, do systematic overestimation or underestimation of tau burden from FDG PET reflect meaningful metabolic signals, such as hypometabolism disproportionate to tau load, or vice versa. If found, that could yield novel insights into the metabolism–tau relationship. Given that the authors have access to longitudinal FDG and tau PET data, exploring how prediction residuals or temporal changes in prediction accuracy relate to disease progression or clinical trajectories could provide novel mechanistic insights that go beyond the prediction framework itself. For example, fast tau-accumulation or low tau-accumulation and its corresponding predictions from FDG-PET at baseline?

We want to thank the reviewer for this constructive suggestion. We agree that exploring the clinical and biological meaning of our model's prediction errors (residuals) significantly elevates the mechanistic value of this work. To address this, we implemented two changes: (1) we replaced the term *"multimodal"* with **"***trans-modal***"** throughout the manuscript to more accurately reflect our framework's translation from one imaging modality to another, and (2) we conducted the suggested post-hoc analyses evaluating baseline prediction residuals (Observed - Predicted FTP-PET) against longitudinal clinical trajectories and downstream tau accumulation.

Our new analyses revealed that baseline model residuals carry significant prognostic value, where baseline residuals significantly correlated with longitudinal changes in the CDR-SB ($P = 0.0004$) and subsequent longitudinal tau accumulation (temporal meta-ROI) ($P = 0.04$). Because the residual is calculated as *Observed minus Predicted*, a positive residual represents individuals whose true tau burden at baseline was underestimated by the FDG-PET metabolic model. Our findings show that these individuals go on to experience significantly faster clinical worsening and more rapid tau accumulation over time.

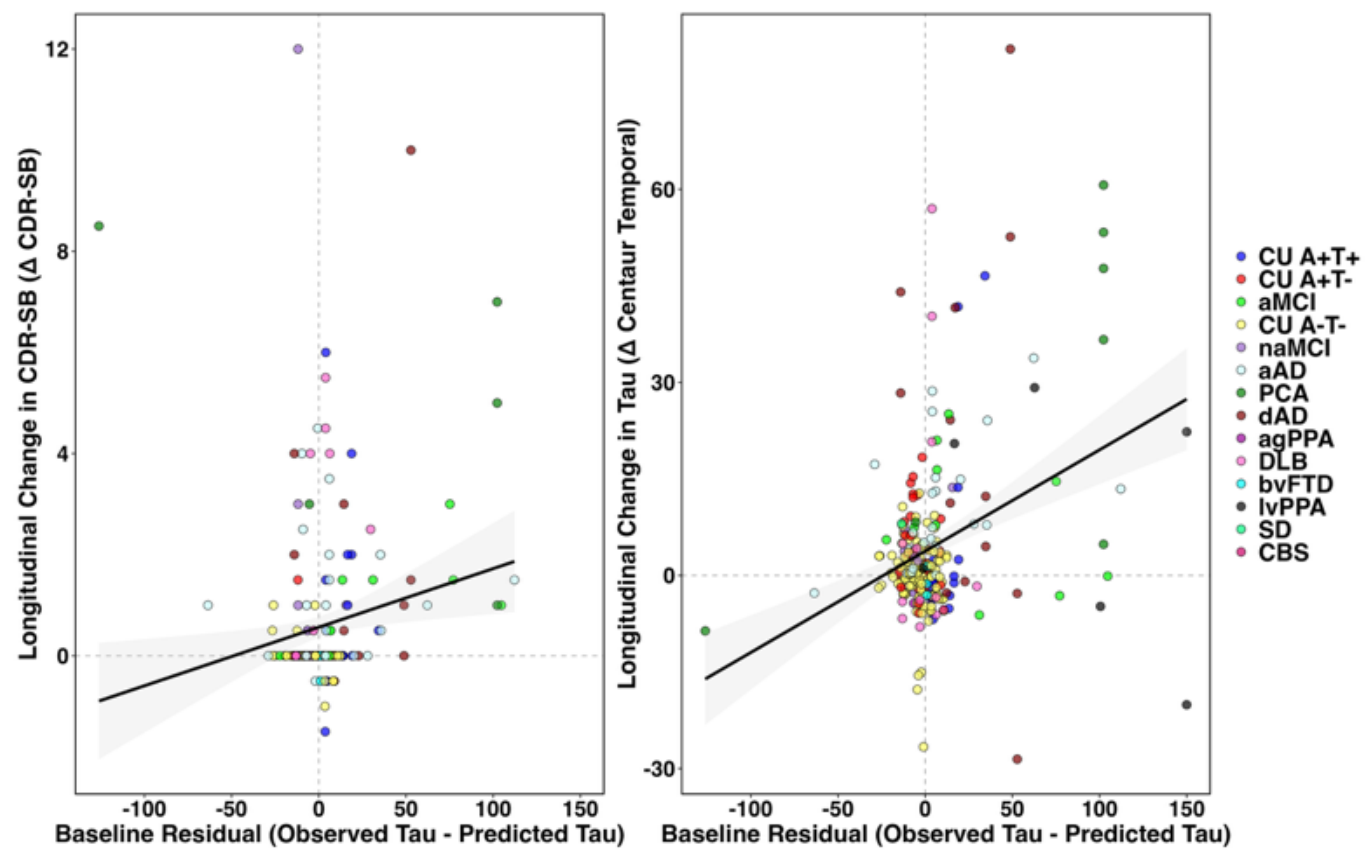

We modified several sections of the manuscript to account for this analysis:

Methods (page 22): *"Finally, we conducted a longitudinal validation analysis to determine whether baseline model prediction errors carry prognostic value for future clinical deterioration and disease progression. For each participant, prediction residuals (observed FTP-PET minus predicted FTP-PET) were calculated for the temporal meta-ROI at the baseline visit. Longitudinal clinical progression and downstream tau accumulation were quantified by calculating the change (Δ) in CDR-SB and the change (Δ) in temporal meta-ROI FTP-PET SUVR between the baseline visit and the final follow-up visit. Because clinical scales and residuals often yield non-normal distributions and tied ranks, the association between baseline prediction residuals and subsequent longitudinal changes (Δ CDR-SB and Δ FTP-PET) was evaluated using partial Spearman rank correlation coefficients. To isolate the specific prognostic effect of initial prediction error from the compounding effect of time, the total longitudinal follow-up duration was statistically forced into the model and controlled for as a covariate."*

Results (page 12): *"Finally, we evaluated whether baseline prediction residuals between predicted and observed FTP-PET in the CenTauR temporal meta-ROI carry prognostic value by assessing their relationship with change (Δ) in CDR-SB and the change (Δ) in CenTauR temporal meta-ROI between the baseline visit and the final follow-up visit for each participant. This yielded a small yet significant relationships between baseline residuals and Δ CDR-SB, $\rho = 0.231$, $P = < 0.001$ and between baseline residuals and Δ temporal meta-ROI, $\rho = 0.125$, $P = 0.04$. This indicates that individuals for which FTP-PET burden was underestimated at baseline tended to clinically worsen and accumulate more tau over time. These relationships are visually depicted in Supplementary Figure 6."*

Discussion (page 17): *"It is also noteworthy that while our model explains a high proportion of variance in patterns of FTP-PET, additional factors may influence inter-individual variability. For instance, post-hoc analyses on baseline residuals and rates of change in CDR-SB and temporal FTP-PET support the notion that variance unexplained by our model carries biological meaning and prognostic value. A potential contributing factor is biological sex, as supported by distinct differences in the proportion of variance explained between females and males in our post-hoc stratified analyses. This aligns with extant literature demonstrating sex-specific effects on trajectories of AD-type tau accumulation that disfavor women.[59] In addition, the model showed slight degree proportional bias in high-tau groups (i.e., dAD, PCA, lvPPA). However, this type of bias for extreme values was expected given the linear assumptions of the model. Future work exploring such factors is needed not only to improve the accuracy of data-driven tools, but also to clarify the mechanistic underpinnings of AD-type tau progression."*

3. Distinguishment between translation and simulation. The authors note that network-based approaches achieved only modest performance in predicting tau burden. However, it is important

to clarify that most such models are designed to simulate tau propagation dynamics from putative epicenters, where individual-level input data serve primarily as constraints on model behavior. These approaches are typically not intended for individual-level prediction. In contrast, the present work performs a cross-modal translation from FDG PET to tau PET, leveraging spatially rich individual-level metabolic information alongside shared vulnerability properties across brain networks. Given these fundamental differences in objective and input structure, the superior prediction performance observed here is expected, and the comparison with network-based simulation approaches should be framed accordingly to avoid a misleading implied equivalence.

We agree with the reviewer's distinction between mechanistic network-based simulation approaches and our framework. We have toned down the language on studies relying on the prion-like paradigm, and removed sentences reporting the R2 values since, as the reviewer points out, the goal and designs are not equivalent. We also modified the Introduction as follows (page 3):

"However, these studies are most often designed to simulate tau propagation dynamics at the region-level and typically not intended to be applied at the individual-level."

And the Discussion (page 16): *"Instead, gradient-based and epicenter-based frameworks evaluate similar questions at different scales, and converge on the notion that large-scale functional systems are central to disease biology, and may even act not only as mere conduits but also drivers of proteinopathies in later life, an aspect often underappreciated in molecular pathology models."*

4. Non-linear prediction approaches. The authors employ linear models for prediction, which yield impressive results and have the advantage of straightforward interpretability. However, the relationship between regional glucose metabolism and tau burden is non-linear, particularly across disease stages and diagnostic categories. It would be informative to compare the current linear approach against non-linear alternatives—such as Random Forest regression, which retains high interpretability via feature importance—or recently developed tabular foundation models such as TabPFN (Hollmann et al., 2025, Nature). If non-linear models yield substantially improved performance, this would be an important finding in itself; if they do not, it would provide useful evidence supporting the linearity assumption and further validate the current approach.

We thank the reviewer for this excellent suggestion. As noted, evaluating non-linear alternatives provides a crucial empirical test of our core modeling assumptions. To address this, we conducted a comprehensive benchmarking analysis comparing our primary linear mixed-effects framework against five widely used regularized and non-linear machine learning architectures: Ridge, Lasso, Elastic Net, Random Forest, and Support Vector Machines (SVM). As summarized in the table below, our Linear model demonstrated the highest out-of-fold cross-validated performance, outperforming both the regularized linear variants and the non-linear machine learning architectures. These findings strongly support our linearity assumption. This aligns with our prior work demonstrating that a linear reduction of metabolic patterns across the ADRD spectrum effectively captures the macro-scale of neurodegeneration compared to non-linear embeddings (Jones et al., 2021).

Supplementary Table 1 Comparison of alternative models in the Mayo research cohort

Model	R^2	MSE	MAE
Linear	0.71 [0.61-0.77]	0.01 [0.01-0.02]	0.09 [0.08-0.09]
Linear with age, sex, and *APOE*	0.71 [0.62-0.78]	0.01 [0.01-0.02]	0.09 [0.08-0.09]
Elastic Net	0.65 [0.54-0.73]	0.13 [0.12-0.14]	0.09 [0.09-0.10]

Lasso	0.65 [0.54-0.73]	0.13 [0.12-0.14]	0.09 [0.09-0.10]
Random Forest	0.59 [0.49-0.59]	0.15 [0.13-0.16]	0.10 [0.09-0.11]
Ridge	0.65 [0.54-0.73]	0.13 [0.12-0.14]	0.09 [0.09-0.10]
SVM	0.64 [0.52-0.72]	0.13 [0.12-0.14]	0.09 [0.09-0.10]
Linear base model*	0.51 [0.40-0.60]	0.13 [0.12-0.14]	0.09 [0.08-0.09]

*Using age, sex, *APOE4* carriership, clinical diagnosis clinical dementia rating scale sum-of-boxes as predictors

We have modified the language to account for these new analyses as follows:

Methods (page 21): *"To evaluate whether non-linear or regularized machine learning architectures could optimize regional FTP-PET prediction relative to our primary linear model, we benchmarked performance against five alternative regression frameworks: Ridge regression (α = 0), Lasso regression (α = 1), Elastic Net regression (tuning both α and λ) Random Forest (implemented via the fast conditional inference forest engine ranger), and Support Vector Machines (SVM) utilizing a Radial Basis Function (RBF) kernel. Each algorithm was trained to predict regional log-transformed FTP-PET SUVR values across participants using the optimal number of FDG-PET eigenbrain eigenvalues based on the linear model. Model optimization and hyperparameters tuning were executed via a 5-fold cross-validation. Out-of-fold cross-validated performance was quantified for every Schaefer-400 region across all algorithms by extracting R^2 values."*

Results (page 11): *"Comparisons with alternative non-linear and regularized models revealed lower performance with R^2 values ranging from 0.59 to 0.65. […] Full performance metrics can be found in Supplementary Table 1."*

Minor Comments:

1. The Mayo research cohort comprises three sub-cohorts acquired across multiple sites. Please clarify whether harmonization procedures (e.g., ComBat or equivalent methods) were applied to account for potential site-related confounds in PET measurement.

We thank the reviewer for raising this important point. Even though ComBat and analogous methods are common in MRI, PET typically use image processing methods where a smoothing kernel for each kernel is estimated to smooth all images to a common resolution. This approach has been used in the current study and is thought to sufficiently reduce inter-scanner variability (Joshi et al., 2009: 10.1016/j.neuroimage.2009.01.057). In addition, recent evidence has shown that ComBaT does not improve ICC performance between scanners in MRI (Gebre et al., 2023: 10.1016/j.neuroimage.2023.119912). Therefore, we opted to keep PET data as is, in line with our previous work using this framework (Barnard et al., 2025: https://doi.org/10.1212/WNL.0000000000213831) and current ADNI practices for FDG-PET (https://doi.org/10.1016/j.jalz.2010.03.003).

2.In the scatter plots of Figures 4, 6, and 7, adding an identity line (y = x) would facilitate visual assessment of prediction bias. Specifically, systematic over or underestimation of tau values, relative to observed measurements.

We appreciate the suggestion. We have added identity lines where appropriate.

3. It would be informative to examine whether prediction performance differs by sex, given the influence of biological sex on AD pathology.

We thank the reviewer for the suggestion. We conducted post-hoc analyses comparing R2 values between males and females, which showed higher values in females. This interesting finding suggests that the coupling between regional glucose hypometabolism and AD-type tau burden

may be tighter in women. This aligns with existing literature indicating accelerated or altered trajectories of tau accumulation in females (Coughlan et al., 2025: doi:10.1001/jamaneurol.2025.0013). We modified the manuscript as follows to account for this analysis and results:

Methods (page 21): *"Models were re-run including age, sex, and APOE status as covariates to assess potential improvement in prediction, and atlas-wide R^2 values were compared across male and female participants."*

Results (page 10): *"However, in order to explicitly assess performance across sexes, R^2 values from the main model were compared between males and females across the Schaefer-400 atlas. This yielded higher values in females (R^2 = 0.75 [IQR 0.66 to 0.81]) compared to males (R^2 = 0.61 [IQR 0.5 to 0.70])."*

Discussion (page 17): *"[…] A potential contributing factor is biological sex, as supported by distinct differences in the proportion of variance explained between females and males in our post-hoc stratified analyses. This aligns with extant literature demonstrating sex-specific effects on trajectories of AD-type tau accumulation that disfavor women.[59] […] Future work exploring such factors is needed not only to improve the accuracy of data-driven tools, but also to clarify the mechanistic underpinnings of AD-type tau progression."*

Reviewer #2 (Remarks to the Author):

This paper presents a machine learning framework for predicting tau-PET burden across the Alzheimer's disease and related disorders (ADRD) spectrum using FDG-PET imaging. The authors leverage StateViewer — a pre-existing clinical decision support system built on principal component decomposition of 1,000 FDG-PET scans — to extract participant-level eigenvalues ("eigenbrains") that serve as predictors in a linear regression model targeting regional tau-PET uptake across the Schaefer-400 atlas. The model was trained on a large Mayo Clinic research cohort (n = 1,069) spanning normal aging, Alzheimer's disease (AD), dementia with Lewy bodies (DLB), and frontotemporal dementia (FTD); a major strength of the study. The optimal model incorporated 50 eigenbrains. Three independent validation experiments on diverse data were conducted, for which the author should be commended. The model demonstrated high training-set performance (median R^2pred = 0.58 at participant level; R^2 = 0.93 at region level) and achieved 84–95% accuracy in tau staging across cohorts.

Strengths:

1. Large training dataset spanning across AD and other neurodegenerative diseases, as well as multiple independent validation sets.

2. FDG-PET is closely related to tau pathology and has great clinical utility. Predicting tau burden and progression using FDG-PET complements the network/epicenter-based framework.

3. The tau staging confusion matrices and the case example in amnestic AD phenotypes (aMCI/aAD) ground the model's value in practical diagnostic scenarios.

We wish to express our gratitude to the reviewer, as their comments have been greatly helpful in improving the manuscript. We have addressed their comments point-by-point, as listed below.

Major comments:

While the authors addressed several important limitations in the manuscript, some of which need to be evaluated.

1. The individual-level prediction is misleading given the model's design and the evaluation metrics. The model is fundamentally a population-level regression framework, not an individual brain model. For each of the 400 Schaefer regions, a separate linear regression is trained across

all 1,069 participants, using their FDG-PET eigenvalues as predictors. When applied to a new individual, population-derived regression coefficients are used to predict where that person falls on the group regression line. Two individuals with identical FDG-PET eigenvalue profiles would receive identical tau predictions, regardless of individual variation in their FDG-PET–tau relationship due to co-pathologies, genetic factors, or metabolic individuality (ie, it is not individual brain modeling in a mechanistic sense).

We completely agree with the reviewer's distinction and appreciate the opportunity to clarify our terminology. Our model is indeed a population-derived regression framework used to estimate individual spatial patterns, rather than a personalized, individual-level simulation. To prevent any misunderstanding, we have carefully revised the manuscript to remove any language implying mechanistic "individual brain modeling." Instead, we explicitly frame the framework as a population-derived translation model capable of individual-level estimation.

Introduction (page 4): "*The model is best framed as a population-level regression framework that leverages shared variance across a large cohort to estimate individual-level spatial distributions of FTP-PET burden.*"

Methods (page 21): *"The FDG-PET-based model of tau prediction is configured as a population-level regression framework. Population-derived regression coefficients are applied to individual participant FDG-PET features to estimate their corresponding regional FTP-PET burden across a standardized brain atlas."*

Results (page 8): *"Models were iteratively tested with an increasing number of eigenbrains, and the optimal model was selected based on the highest overall leave-one-out R^2_{pred} value. This was applied both across participants (i.e., using participant-level values) and region-level (i.e., by aggregating participant values across Schaefer-400 regions)."*

2. The paper repeatedly describes its R^2 values as "participant-level" metrics, but this label is misleading. What is actually being computed is, for each brain region, the proportion of variance in tau across participants explained by the model, with the median taken across the 400 regions (IQR reflecting variability across regions). The R^2pred = 0.58, therefore, tells the reader: across 400 regions, the median proportion of variance in tau levels across participants explained by the model is 58%. This is a population-level cross-sectional correlation, not an index of how accurately the model predicts any given individual's tau distribution. True individual-level evaluation would require computing, within each participant, the spatial correlation between their 400-element observed tau vector and their 400-element predicted tau vector — an individual-level spatial R^2 across brain regions. The authors should show the distributions of individual-level correlations and MSEs, or at the very least adjust the tone of individual predictions across the results and interpretation/discussion.

We thank the reviewer for this statistical clarification. We agree that our original framing conflated across-participant regional model performance with true within-subject spatial accuracy. To evaluate the model's performance at the individual level, we performed the reviewer's suggestion. Specifically, for each training/validation cohorts, we computed participant-level spatial R^2, MAE, and MSE across their 400-element observed and predicted tau vectors. Our results demonstrate that the trans-modal framework preserves intra-individual spatial distribution fidelity, with R^2 ranging from 0.72 to 0.80. Of note, we removed the region-wise analyses to avoid redundancy, and because the participant-level spatial regressions seemed more relevant to report.

We have updated the manuscript text to distinguish between these two complementary evaluation dimensions (across-participant vs. within-subject spatial R^2) and have added spatial correlation R^2 in Figures (aggregated across groups). We also modified the text as follows:

Methods (page 21): *"In addition, we computed spatial R^2 values between observed and predicted FTP-PET across the Schaefer-400 atlas within each participant, and report the median value aggregated across the cohort."*

3. Another substantive methodological problem in the paper is the overlap between the longitudinal validation cohort and the training set. A re-analysis using only participants whose baseline data were withheld from training would provide a cleaner estimate of temporal generalizability. At least, a sensitivity analysis that excludes longitudinal participants from the training set would be helpful.

We completely agree with the reviewer's critique and completed the suggested sensitivity analysis. While marginal R^2 was reduced, classification results remained unchanged after using a training model without individuals with longitudinal follow-up. We have modified the text to account for this sensitivity analysis:

Methods (page 22): *"Sensitivity analyses were performed where baseline-trained models of FTP-PET prediction were re-run after excluding participants who had longitudinal data available. These models were subsequently used to replicate the longitudinal validation analyses listed above. This was done to assess whether any overlap between the training and validation cohorts influenced predictive performance in the longitudinal analyses."*

Results (page 12): *"Analyses were repeated using a baseline training model without participants with longitudinal data to assess whether overlap between the training and validation set influence performance. Performance of mixed-effect linear models across the Schaefer-400 atlas across participants yielded lower performance, with a marginal R^2 = 0.39 [IQR 0.33 to 0.46], MAE = 0.03 [IQR 0.03 to 0.03], and MSE = 0.002 [IQR 0.001 to 0.002]. Performance of mixed-effect linear models across the CenTauR regions yielded marginal R^2 = ~0.33 to 0.47, MAE = ~0.06 to 0.07, and MSE = ~0.007 to 0.009. However, this had no impact on tau staging classification results, which remained identical as the original model."*

4. As mentioned in the study, the tau-PET tracer used does not typically bind to non-AD tauopathies. It's not clear what the meaning of applying tau stages to non-AD cases is.

We thank the reviewer for this critical point. We completely agree that the tau-PET tracer used typically shows negligible binding to the straight tau filaments (3R/4R) characteristic of non-AD tauopathies. The application of AD-tau staging to clinically diagnosed non-AD cases serves two purposes: (1) By including primary non-AD neurodegenerative cohorts, we establish that our model does not simply map generic regional neurodegeneration or translate regional FDG-PET hypometabolism into spatially equivalent FTP-PET stages. Instead, it accurately distinguishes etiologic-specific patterns; (2) it is well-established that patients presenting with clinical syndromes typically associated with non-AD etiologies, such as bvFTD or CBS, can harbor significant underlying AD co-pathology, or have their symptoms driven entirely by an atypical presentation of AD. As demonstrated in Table 1, a significant proportion of our non-AD clinical cohort was indeed amyloid- and tau-positive on biomarkers.

To address this explicitly (in tandem with our response to Reviewer 1), we conducted additional analyses specifically targeting these challenging, heterogeneous clinical groups (bvFTD and CBS). Our model achieved high classification accuracy (81–85%) for tau staging in these groups, successfully avoiding the prediction of an elevated FTP-PET burden in cases dominated purely by FDG-PET hypometabolism without underlying AD tau. These findings are now detailed on page 11 and visualized in Figure 4.

In addition, we have amended the Discussion (page 16) to explicitly clarify this interpretation for the reader:

“This also supports the model’s specificity to AD-type tau in that predictions are driven by specific patterns of whole-brain metabolism rather than simply translating regional FDG-PET signal into spatially equivalent FTP-PET patterns.”

“This suggests that our model could potentially (1) identify “off-diagonal” symptomatic individuals with possible symptom-driving co-pathologies, and (2) identify rare AD phenotypes that are traditionally caused by non-AD etiologies.”

5. FDG-PET hypometabolism is likely largely a downstream consequence of tau-related neurodegeneration in AD. The model is framed as predicting tau from FDG-PET, but the causal relationship likely runs in both directions, or from tau to hypometabolism. The claim that FDG-PET gradients constitute a “blueprint” for tau accumulation, rather than a mirror or downstream effect of it, is a strong causal inference that the cross-sectional correlational design cannot support.

We agree with the reviewer that this was strong-worded. We modified this part of the Discussion which now reads as follows (page 16):

“Instead, gradient-based and epicenter-based frameworks evaluate similar questions at different scales, and converge on the notion that large-scale functional systems are central to disease biology, and may even act not only as mere conduits but also drivers of proteinopathies in later life, an aspect often underappreciated in molecular pathology models.”

6. Figs 3 and 6. It would be more interesting to show the real/observed tau-PET maps (or differences) alongside the predicted maps across the cohorts.

We have modified Figure 3 and 6 to display group-wise observed and predicted renderings side-by-side, along with median spatial R^2 values for each pair.

7. Fig 5A. It is not clear what the subject-level error in prediction (qualitatively and more importantly quantitatively).

We now report marginal R^2, MAE, and MSE for all analyses, and have added the marginal R^2 on Figure 5.

Minor:

8. The abstract results are a bit vague in some instances without quantification or details, eg: “The model accurately predicted both the magnitude and spatial distribution of taupathology at the individual and regional levels across cohorts” Or “generation of biologically plausible tau patterns in a clinical cohort without tau-PET”

We have made clarifications to the abstract and added quantitative metrics where possible.

9. Figure 2B shows a greater hypometabolism towards more red color. But more hypometabolism is associated with lower FDG-PET SUVR values. Either inverse the color or change the colorbar scale label to hypometabolism rather than FDG-PET SUVR.

We have modified the color scheme and specifically added “hypometabolism” and “normal metabolism” labels to the color bar.

10. Fig 4A, ideally results for more ROIs should be shown and their corresponding correlations/R^2. B) What is the recall/precision of the model for this staging?

We now show 6 regions exampled on Figures 4, 6, and 7, and report all performance metrics of confusion matrices either in-text or in Supplementary Tables.

11. No stats are included in the majority of the figures/figure captions

Statistics have been added to most Figures, where applicable.

12. There are multiple brains overlapping in the Eigenbrain 7 in Supp Fig1.

Thank you for catching this. We have made the correction.

Reviewer #3 (Remarks to the Author):

The manuscript by Corriveau-Lecavalier et al describes an FDG-PET and brain function informed machine learning model to predict regional tau-PET SUVRs across a range of older adults, individuals with AD at different clinical levels of severity, and individuals with non-AD neurodegenerative diseases. The introduction is well-written, and many of the relevant papers are cited. The description of brain networks, gradients, and their relationship to function and cognition are appropriate. As someone who was not familiar with StateViewer before reading this manuscript, I nonetheless found the methods (and Figure 1) sufficiently clear. The manuscript uses independent samples for replication and a “longitudinal” replication as well, and they correctly acknowledge the limitations of this latter sample. I think the statistical analyses are largely appropriate, however, I was left with many questions about model performance and characteristics that were unanswered (see below). These made it difficult to evaluate some of the study’s conclusions. Overall, their model shows FDG-PET can predict late-stage tau pathology measured by [18F]flortaucipir, with regional patterns of flortaucipir uptake that correspond well to known uptake patterns in a number of different AD syndromes (aAD, dAD, PCA, lvPPA). I have a few concerns, as well as some minor suggestions further below.

We thank the reviewer for their comments. Their suggestions significantly enhanced the paper.

Major:

Missing from this model is replication with a non-flortaucipir tau-PET tracer (I acknowledge that such a dataset with FDG-PET and a non-FTP tau-PET tracer may not exist). Because of flortaucipir’s lower sensitivity to tau and higher levels of noise (Ghatamaneni et al Acta Neuropathologica 2025), one wonders about whether FDG is able to capture the broader dynamic range of tau pathology that can be captured with other tau-PET imaging agents. In this connection, FDG may be better-suited in predicted tau that has reached the detection threshold of Flortaucipir, but perhaps less useful at detecting lower levels, which is especially relevant in preclinical disease. On a related note, some tau-PET agents such as PI2620 have been reported to bind to non-AD tauopathies, especially PSP 4R tau and to a lesser extent CBD 4R tau. How do the authors suggest their model would generalize under these circumstances? Perhaps taking these limitations together, if a dataset without FDG and a different tau-PET ligand does not exist, it would be better to call the maps “predicted Flortaucipir maps” rather than tau-PET, as some elements of the results may not generalize to other tau-PET ligands.

We thank the reviewer for this constructive point. We completely agree that different tau-PET tracers exhibit unique dynamic ranges, signal-to-noise ratios, and binding affinities. As the reviewer correctly notes, a multi-tracer dataset containing paired FDG-PET and non-FTP tau-PET imaging was not available to us for this study. To address this limitation and prevent over-generalization, we have taken two steps: (1) we have followed the reviewer's suggestion and systematically modified our language throughout the manuscript. We have replaced general terms like "tau-PET" with "FTP-PET" to accurately reflect the specific tracer profile our model was trained on; (2) we have expanded our Discussion to explicitly mention that our model applies to FTP-PET and that future work is needed in a broader range of ligands (page 17):

“Sixth, our model was only trained on FTP-PET imaging, and it remains to be tested whether it would perform similarly using different ligands with a broader dynamic range, or even recent ligands that may bind to primary tauopathies such as PI2620.”

Regarding the study's main conclusions, can the authors please elaborate on how this study advances the mechanistic insight into neurodegeneration and tau-PET (mentioned in abstract and discussion)? I agree with the authors that some pathologies accumulate preferentially within specific networks/gradients, and that some clinical syndromes are more likely to be associated with specific pathologies. But I don't see how the present manuscript's model helps explain anything mechanistic about vulnerability/resilience of any specific network. Because of the large amount of neurodegenerative tauopathies in which there is an FDG-Flortaucipir dissociation (~40% of bvFTD, ~50% of CBS, most PSP, most agPPA cases are 3R or 4R tauopathies), I do not understand how the model provides mechanistic insights into the relationship between tau and neurodegeneration.

We thank the reviewer for this insightful critique. We agree that the term "mechanistic" requires precise contextualization, especially given the known dissociation between FDG-PET hypometabolism and flortaucipir retention in non-AD primary tauopathies (e.g., 3R or 4R tauopathies like bvFTD, CBS, and PSP). To address this, we have refined our language throughout the manuscript to explicitly refer to "AD-type tau" (3R/4R paired helical filament tau) rather than tau neuropathology in general. Our model is specifically optimized to capture the relationship between FDG-PET (as a proxy for neurodegeneration) and this specific AD-type tau conformation.

Mechanistically, our work advances the field by shifting the framework from localized focal progression to macroscale systemic vulnerability. The dominant paradigm in AD has focused heavily on the prion-like spreading hypothesis, which emphasizes localized tau epicenters and trans-synaptic seeding. In contrast, our model demonstrates that AD-type tau accumulates along large-scale, continuous metabolic gradients. These gradients encapsulate intrinsic biological properties supporting brain function, such as gene expression profiles, neurotransmitter receptor densities, and metabolic demands. By showing that tau topology aligns with these pre-existing functional axes, our study provides macroscale mechanistic insight into why certain distributed networks are inherently vulnerable or resilient to AD pathology. While this gradient-based vulnerability framework has gained significant traction in cognitive and computational neuroscience, its explicit application to AD proteinopathies represents a novel contribution of our manuscript.

Another important missing element, in my opinion, is the ability to predict tau in more clinically/radiographically challenging scenarios: In CBS, for example, where AD accounts for ~50% of CBS cases, how can an FDG-based model predict which cases are caused by AD-type tau? This is a clinically important question that remains unaddressed. CBS is not included in the present study, but CBS data is reported in the Stateviewer manuscript, and I wonder why these results are not reported.

We completely agree with the reviewer that examining our model within clinically heterogeneous populations and non-AD groups is important. To address this, we have added a group of 22 individuals with CBS. We also conducted additional analyses in this group as well as bvFTD (as both can be caused either by AD or other primary etiologies) and aMCI/aAD, where symptom-driving co-pathologies are common (e.g., TDP-43 or LATE). Our model achieved strong classification accuracy (82-85%) for tau staging within these challenging groups (this reached 92% when only considering bvFTD and CBS who are A+), demonstrating that it successfully avoids predicting a high FTP-PET burden in non-AD disorders despite the presence of FDG-PET hypometabolism. These findings are detailed on page 11 and visualized on Figure 4.

Can the authors show side-by-side display of predicted vs observed tau-PET in the training and validation cohorts? As currently displayed, while Figure 3 recapitulates expected relationships between neurodegenerative clinical syndromes and patterns of tau-PET, without seeing the

observed data, these brains are not informative. While I appreciate that Supplementary Figure 3 shows this in scatterplot format, I suggest reporting R2 values for each predicted-observed pair. Perhaps even better could be displaying R2 brain-maps. Bland-Altman plots showing the difference between predicted and observed tau-PET, with colours highlighted similar to figure 4 would be helpful to present.

We thank the reviewer for these suggestions, which have been highly beneficial for improving our understanding of our model's behavior. We have made the following modifications to the manuscript: (1) observed versus predicted pairs are now displayed side-by-side in Figures 3 and 6 along with median spatial R^2 values; (2) Supplementary Figure 3 has been added to display R^2 values across participants on brain renderings; and (3) Supplementary Figure 4 has been added to show region-wise Bland-Altman plots for each group, color-coded by Yeo's networks. This showed *that while the overall mean predicted values generally fall within the 95% limits of agreement of the observed values, high-tau groups (i.e., aAD, dAD, and lvPPA) exhibit a slight degree proportional bias, characterized by overestimation at lower mean values and underestimation at higher mean values. However, this was expected given the linear assumptions of our model. Moving forward, we aim to explore the factors explaining these predictive differences across clinical phenotypes as we advance toward clinical implementation alongside our StateViewer work.*

We have modified the text to account for this as follows:

Methods (page 21):

"Visual inspection of model performance included brain renderings of R^2 values across participants in each group, and Bland-Altman plots across the Schaefer-400 atlas to assess systematic over- and under-estimation of FTP-PET values at the group- and region-level."

Results (page 8): *"Supplementary Figure 4 shows Bland-Altman plots across all diagnostic groups. While the overall mean predicted values generally fall within the 95% limits of agreement of the observed values, high-tau groups (i.e., aAD, dAD, and lvPPA) exhibit a slight degree proportional bias, characterized by overestimation at lower mean values and underestimation at higher mean values."*

Discussion (page 17): *"In addition, the model showed slight degree proportional bias in high-tau groups (i.e., dAD, PCA, lvPPA). However, this type of bias for extreme values was expected given the linear assumptions of the model."*

Moreover, can the authors report observed vs predicted tau-PET in the medial temporal (perhaps entorhinal cortex and amygdala) and temporal meta-ROI for the ADNI and Mayo cohorts?

Unfortunately, the Schaefer-400 atlas only includes cortical regions, and the CenTauR atlas does not offer this level of granularity of the mesial temporal lobe. However, we modified Figures 4, 6, and 7 to include all CenTauR regions, including the mesial temporal lobe.

The authors combined stages A&B and stages C&D because of class imbalance problems, which is understandable. I am concerned that this creates an artificially "easy" task of distinguishing between those with little-to-no detectable tau vs those with moderate-to-severe tau.

We appreciate the reviewer's concern regarding the granularity of the staging model. We agree that predicting a dichotomized stage (A/B vs. C/D) is statistically less complex than predicting a four-class model (A vs. B vs. C vs. D). However, this grouping was strictly necessitated by severe data constraints and is strongly supported by current clinical practice; In our training cohort, intermediate stages were highly underrepresented in A+ individuals, with only 6.46% of individuals classified as Stage B and 9.34% as Stage C. Attempting to train or validate a machine learning model on these severely underpowered intermediate categories would lead to highly

unstable predictions and overfitting. In addition, separating individuals into "low/medium" vs. "high" tau burdens directly mirrors current clinical and regulatory realities. For instance, recent clinical trials for anti-amyloid therapies, most notably Donanemab, explicitly dichotomized patient populations into low/medium vs. high tau sub-populations to determine treatment efficacy and eligibility. Therefore, our model's classification design is highly relevant to contemporary clinical decision-making.

To transparently address the lack of intermediate tau cases, we have added this limitation to the Discussion (page 17): *"In addition, there was evidence of class imbalance in tau stages, with a minimal proportion of cases presenting with intermediate tau stages (B/C). This warrants validation in future cohorts featuring a more evenly distributed representation of tau stages."*

The results mention that the FDG-PET model was not improved by the addition of covariates. However, I wonder how much better this advanced model is compared to a base model of age, sex, APOE4 genotype, clinical diagnosis and MOCA/CDR-SoB.

This is a great suggestion. We now benchmark our model against a base model including age, sex, *APOE4* carriership, clinical diagnosis, and CDR-SB as predictors of FTP-PET instead of FDG-PET. This yielded R^2 = 0.51, which is lower than our main model. These new benchmarking analyses demonstrate that the complex spatial information captured by our FDG-PET framework provides substantial predictive power beyond traditional clinical and demographic variables. These findings are now integrated into the Methods and Results sections, and detailed in Supplementary Table 1.

Methods (page 21): *"The optimal model was additionally benchmarked against a base model using age, sex, APOE4 carriership, clinical diagnosis, and CDR-SB as predictors of FTP-PET across participants."*

Results (page 11): *"The base model including age, sex, APOE carriership, diagnosis, and CDR-SB also achieved lower performance, with an R^2 value of 0.51."*

The authors elected not to try to predict tau in A- individuals. However, to me, this misses an important opportunity for showing the model predicts "true negatives". It is important to see how the FDG-PET and brain function model can predict tau-PET stages in non-AD diseases, and the accurate prediction of little-to-no tau-PET (here referring to 3R&4R PHF tau) is a highly relevant clinical scenario (I suspect based the model will be able to do this well). Can the authors please report the results of these analyses?

We appreciate the reviewer's comment. Technically, the stage "A-" is determined by amyloid status only, not by tau. This means that individuals with a high tau burden would remain A- if their Centiloid value is below the 25 threshold.

To address this explicitly (in tandem with our response to Reviewers 1 & 2), we conducted additional analyses specifically targeting challenging, heterogeneous clinical groups that can be caused either by AD or primary tauopathies (bvFTD and CBS). Our model achieved high classification accuracy (82–85%) for tau staging in these groups, successfully avoiding the prediction of an elevated FTP-PET burden in cases dominated purely by FDG-PET hypometabolism without underlying AD tau.

These findings are now detailed on page 11 and visualized in Figure 4. We also added Supplementary Figure 6 showing these data across all groups, demonstrating that predicted tau in A- individuals was low across the board.

We have amended the Discussion (page 16) to explicitly clarify this interpretation for the reader:

"This also supports the model's specificity to AD-type tau in that predictions are driven by specific patterns of whole-brain metabolism rather than simply translating regional FDG-PET signal into spatially equivalent FTP-PET patterns."

"This suggests that our model could potentially (1) identify "off-diagonal" symptomatic individuals with possible symptom-driving co-pathologies, and (2) identify rare AD phenotypes that are traditionally caused by non-AD etiologies."

Can the authors please state whether any other individual-level data was used in the model? In the methods, I think the explanation of how the FDG-PET data are used in the model is well-explained, however, I am less clear about the model uses brain function: does this refer to the relationship of FDG-PET signal mapping onto brain networks/gradients associated with cognition and behaviour?

No other individual-level data was used in the model. To clarify how the FDG-PET data maps onto these spatial gradients and constitutes the model's primary input, we have updated the Methods section (page 20):

"Each eigenbrain represents an orthogonal axis of spatial metabolic variation across the cortex, ordered by the proportion of variance explained, and each eigenvalue quantifies the degree to which an individual's metabolic pattern aligns with the spatial topology of that eigenbrain. As such, each participant-level eigenvalue for each eigenbrain represents a mapping onto a specific FDG-PET gradient, resulting in a 1088 X 150 (participant X eigenvalue) table as input."

Figure 4B: Can the authors please present a similar plot for MTL tau? Also, because many readers (myself included) do not yet have a strong intuition regarding CenTauR values, can you please display FTP SUVRs on the right y-axis?

As mentioned above, the Schaefer-400 atlas only includes cortical regions. We modified Figures 4, 6, and 7 to include all CenTauR regions, including the mesial temporal lobe.

Figure 5: In my view, the analyses on stage progression, are interesting and important; I just have a hard time interpreting them without knowing more basic performance. Can you please display a 4x4 matrix with baseline observed vs predicted tau-PET stages? Can the manuscript report overall accuracy of stage transitions that do not include the A- group? The inclusion of the large A- group can artificially increase accuracy statistics.

We have added the baseline confusion matrix to Figure 5, and now report all comparisons with and without including A- individuals. We also report full performance metrics in Supplementary Tables 2-5.

Is it possible the R2 statistics are inflated by the large proportion of CU individuals, who have very little to no tau-PET?

We have added Supplementary Figure 3, which shows R2 values across participants, stratified by group across the Schaefer-400 atlas. This shows markedly higher values in groups with a wider FTP-PET dynamic range (e.g., dAD, PCA) than groups with minimal FTP-PET signal (e.g., CU groups, SD, PSP), supporting the fact that the CU groups did not drive the statistics. In fact, re-running the model without CU groups yielded higher performance, with R^2 of 0.74, adjusted R^2 or 0.69, and predicted R^2 of 0.63.

Minor:

Please mention in the limitations that ADNI is heavily pre-screened and has a much lower level of co-pathology than other cohorts. It would be interesting to see replication in a more diverse setting.

We agree with the reviewer. In fact, this very aspect of ADNI motivated us to include the ADTC cohort, which, albeit being biased due to the highly specialized clinical settings, mitigates this concern. We have added this statement to the limitations (17):

"Seventh, it is important to note that the ADNI cohort is heavily pre-screened for AD-centric symptoms and biology, and is minimally representative of real-world clinical populations. While the inclusion of the ADTC cohort mitigates this to some degree, replication in larger, clinically heterogeneous cohorts is warranted."

Supplementary Fig 2: Perhaps I have misunderstood, but I do not see where or how the Yeo 17 network masks are evaluated/displayed.

We have added a Y-axis title indicating that rows are Yeo's networks.

Supplementary figure 3: Can the line of identity please be shown to facilitate interpretation?

We appreciate the suggestion. We have added identity lines where appropriate.
